# The origin of the particle-size-dependent selectivity in 1-butene isomerization and hydrogenation on Pd/Al_2_O_3_ catalysts

**DOI:** 10.1038/s41467-021-26411-8

**Published:** 2021-10-20

**Authors:** Alexander Genest, Joaquín Silvestre-Albero, Wen-Qing Li, Notker Rösch, Günther Rupprechter

**Affiliations:** 1 Institute of Materials Chemistry, Technische Universität Wien, Getreidemarkt 9/BC, A-1060 Vienna, Austria; 2 Institute of High Performance Computing, Agency for Science, Technology and Research, 1 Fusionopolis Way, #16-16 Connexis, Singapore, 138632 Singapore; 3 Department Chemie and Catalysis Research Center, Technische Universität München, D-85747 Garching, Germany; 4 Present Address: Laboratorio de Materiales Avanzados, Departamento de Química Inorgánica-IUMA, Universidad de Alicante, E-03690 San Vicente del Raspeig, Spain

**Keywords:** Heterogeneous catalysis, Chemical engineering, Porous materials

## Abstract

The selectivity of 1-butene hydrogenation/isomerization on Pd catalysts is known to be particle size dependent. Here we show that combining well-defined model catalysts, atmospheric pressure reaction kinetics, DFT calculations and microkinetic modeling enables to rationalize the particle size effect based on the abundance and the specific properties of the contributing surface facets.

## Abstract (web summary)

The selectivity of Pd catalysts in 1-butene hydrogenation and isomerization depends on nanoparticle size. Herein, the authors show by combining experiment and modeling that the type and specific properties of the contributing surface facets are decisive for the selectivity.

## Introduction

**Fig. 1:**
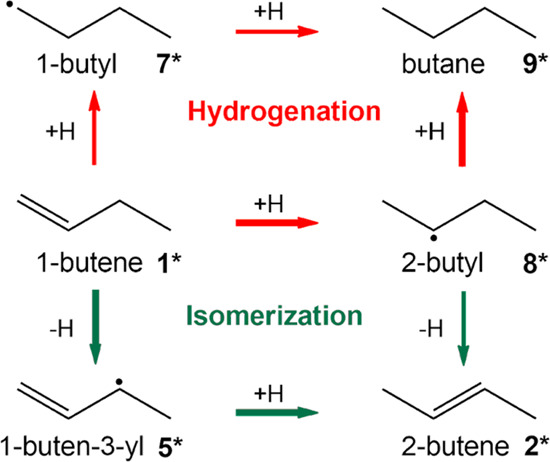
Reaction network for 1-butene isomerization, green, and hydrogenation, red, on Pd catalysts. Hydrocarbon species are identified using bold numbers consistent with ref. ^8^.

Olefinic hydrocarbons constitute valuable key building blocks in the chemical industry, that are used for a wide range of products, including solvents, coatings, and polymers^1–5^. Selective hydrogenation and hydroisomerization are thus critical steps in upgrading refinery streams. Butenes are a distinct example: 2-butenes and isobutene are desirable for high octane gasoline, whereas 1-butene is important in polymer chemistry^6^. In view of the recently increased price of olefinic hydrocarbons (resulting from the “olefin gap”), there is a strong industrial interest in manufacturing specific isomers^6^. This highlights the importance of selectivity when carrying out isomerization, while avoiding hydrogenation to less valuable products. Note that both types of transformations involve hydrogen addition and are catalyzed by the very same Pd/Al_2_O_3_ catalyst. Hydrogenation and isomerization of 1-butene may proceed via a common (hydrogenated) 2-butyl intermediate^7,8^, but isomerization is also possible via an initial dehydrogenation, that does not directly facilitate hydrogenation, cf. Fig. 1. Based on a recent combined experimental and theoretical study of Pd single crystals, the second route was identified as being preferred^9^. The catalyst performance can be tuned via the size dependent properties of the metal nanoparticles, that affect reactivity in different ways. The size-dependent electronic structure of clusters and nanoparticles has been intensively discussed^10–19^, but the combination of specific facets (characterized via their generalized coordination number) is equally important.^20–34^.

In this work, based on 1-butene as example, we study the effects of particle size on both kinds of reactions, hydrogenation and isomerization. We combine experiment and modeling to discern factors influencing both routes using well characterized model catalyst particles. A crucial factor to understand the selectivity will be the type and roughness of the Pd nanoparticle facets.

## Results

### Pd/Al_2_O_3_ model catalysts

To examine the selectivity of the catalyst, we resorted, once again, to a “surface science” model catalyst approach, combined with high pressure cell kinetic tests and theoretical modeling, for details see Supplementary Notes 1–3 of the Supplementary Information (SI). Pd nanoparticles (NPs) were grown impurity-free on Al_2_O_3_ model supports under clean ultrahigh vacuum (UHV) conditions, with exact control over the nucleation density (number of Pd NPs/cm^2^; via the substrate temperature) and the Pd amount (number of Pd atoms/cm^2^; via the evaporation dose measured by a quartz microbalance). Thus, the mean Pd particle size (or the number of Pd atoms/NP) in the systems under study is well defined.

The structural characteristics of the support and the Pd NPs have previously been examined in detail by H.-J. Freund’s group using an array of surface-sensitive methods (see the SI)^35–40^. Here, we deliberately prepared Pd NPs with a mean size of 2–8 nm, so that all NPs essentially exhibit bulk electronic structure, i.e., they are in the “scalable” regime^10,15–17^. Thus, variations of the electronic structure, that small Pd clusters (with less than 80 atoms) may exhibit^16^, are not to be expected. Given that Al_2_O_3_ is a rather inert support, metal/oxide boundary effects may be largely excluded, so that the reactivity can be solely assigned to the Pd NPs; the pure support did not show any reactivity.

**Fig. 2:**
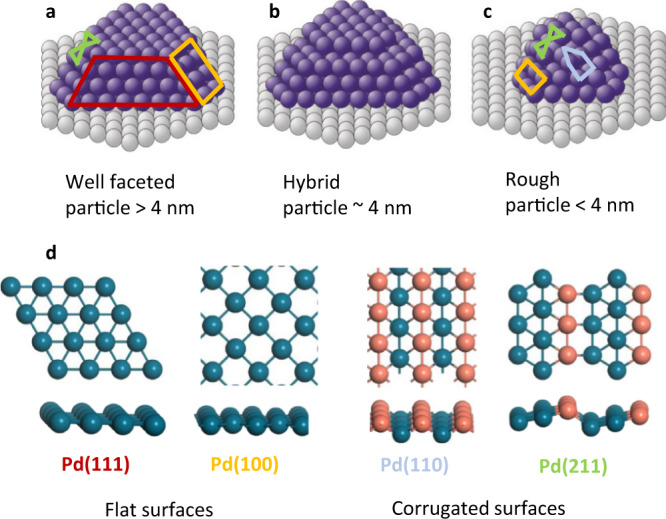
Schematics of Pd nanoparticles as catalysts by combining smooth and stepped single crystal surfaces. **a** Large particles carry extended patches of smooth surfaces, modeled by selected facets. **b** “Hybrid” particle of larger size with rougher surface. **c** Smaller rough nanoparticle. **d** Ball and stick models of the smooth surfaces Pd(111) and Pd(100), and the rougher surfaces Pd(110) and Pd(211).

Resulting from the current preparation conditions, Pd nanocrystals larger than 4 nm are well-developed truncated cuboctahedra (according to scanning tunneling microscopy (STM))^41–44^, mainly exposing (111) and (100) surface facets (Fig. 2a; further characterized by infrared (IR) spectra of adsorbed hollow, bridge and on-top CO^38,45–47^, agreeing well with corresponding wet-chemically prepared catalysts)^48–51^. The low-index facets may exhibit few steps and are terminated by edges. The Pd nanocrystals with a mean size below 4 nm typically show round/irregular outlines in STM and have rough stepped surfaces (cf. Fig. 2c; again confirmed by stronger or even dominating on-top CO peaks in IR). For preparation and structure details, see Supplementary Note 3 of the SI.

### Kinetic tests at atmospheric pressure

After UHV preparation, the various Pd/Al_2_O_3_ model catalysts (mean NP diameter from 2 nm to 8 nm) were transferred in UHV to a “high-pressure” reaction cell^52^, where batch reactor kinetic studies of 1-butene isomerization and hydrogenation were carried out at atmospheric pressure. Reaction conditions were chosen to avoid β-Pd-hydride formation. Reactant and product analysis by gas chromatography as a function of reaction time enabled us to determine catalytic rates (turnover frequencies) and selectivity over ensembles of specific, rather uniform particle sizes. Given that only weakly adsorbing reactants are used, we do not expect restructuring of the particles as seen in CO or oxygen or under high vacuum^53,54^, especially not within 10 min after which we evaluated the TOFs. For details of the kinetic measurements, see the SI.

**Fig. 3:**
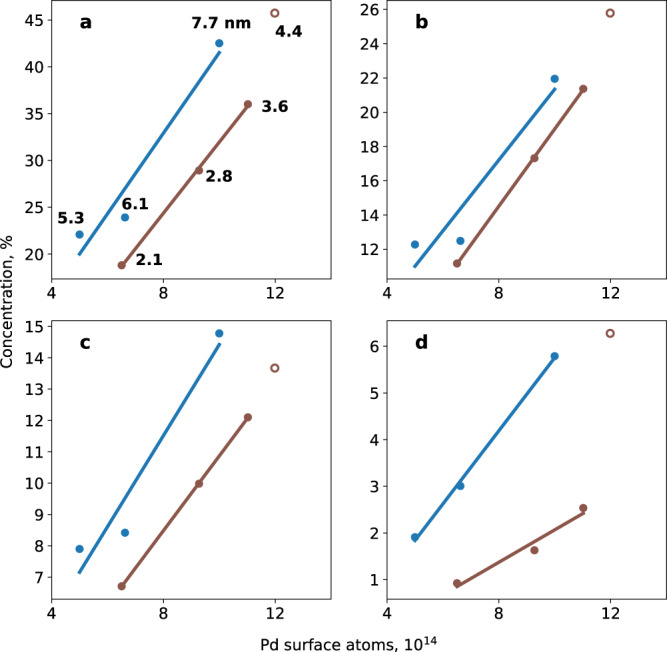
Composition of the gas phase in the reaction cell at 373 K after 10 min. Isomerization and hydrogenation of 1-butene on various Pd/Al_2_O_3_/NiAl(110) model catalysts, over NPs with smooth (blue) and rough (brown) surfaces; average particle diameters (nm) indicated in panel **a**. Shown are the fractions (in percent) as a function of the total number of Pd surface atoms: **a** converted reactant 1-butene, as well as products **b** trans-2-butene, **c** cis-2-butene, and **d** n-butane; see also the SI. Also shown are the results for NPs of 4.4 nm diameter, in between both regimes (brown circles; see text). Initial state: P_1-butene_:5 mbar; P_H2_:10 mbar; Ar added to 1 bar.

Figure 3 shows the 1-butene conversion as a function of the total number of Pd surface atoms in the catalyst samples, exactly known from combining microbalance and STM data. For both the small/rough Pd particles (Fig. 3a, brown line) and the larger/faceted Pd particles (Fig. 3a, blue line), the conversion of 1-butene scales linearly with the number of Pd surface atoms, but the larger particles are still more active, as shown by a constant shift of ~8 %, that clearly indicates an effect of surface roughness. The same holds true for the yields of trans-2-butene (Fig. 3b), cis-2-butene (Fig. 3c) and n-butane (Fig. 3d), although for the latter the notably different slopes indicate variations in the selectivity ratio, depending on the surface roughness.

**Fig. 4:**
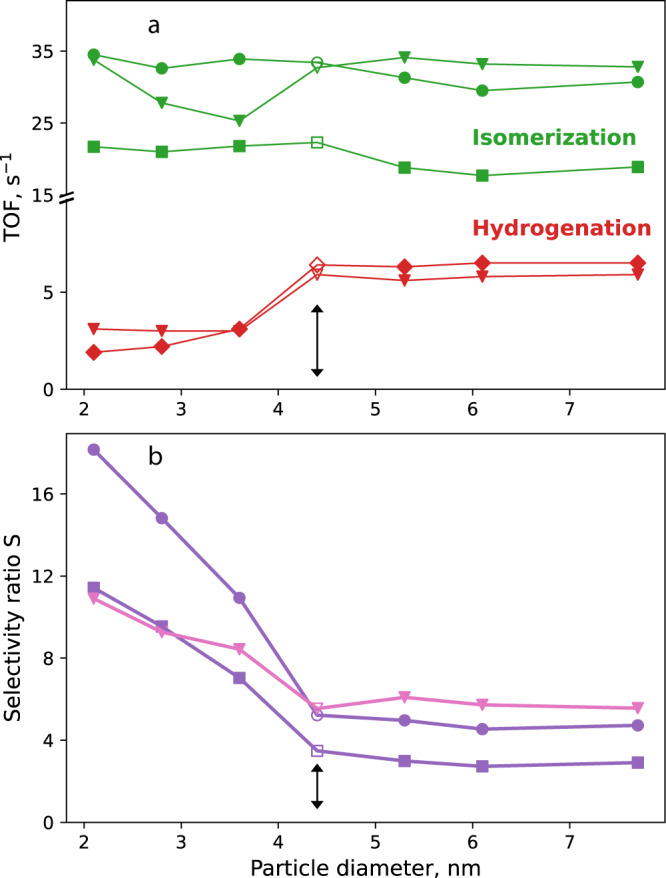
Catalytic performance of Pd/Al_2_O_3_/NiAl(110) catalysts as a function of the average size of the Pd NPs. **a** Turnover frequencies (TOFs) of isomerization (green) and hydrogenation (red) of 1-butene at 373 K and **b** selectivity ratio *S*, of TOFs of isomerization vs. hydrogenation from experiment (purple) or modeling (pink). Data points from experiment (trans + cis-2-butene—discs, trans-2-butene—squares, n-butane—diamond), and modeling (inverted triangles); special case of 4.4 nm labeled by empty symbols, all other data represented by solid markers. Double arrows indicate the trend change in selectivity ratio. Modeling of isomerization focused on the *trans*-2-butene isomer.

Figure 4a displays the particle size dependence of the turnover frequencies (TOFs, equivalent to the corresponding reaction rates measured/calculated after 10 min) per Pd surface atom: for details, see the SI. The isomerization of 1-butene (green markers) shows only a very weak size dependence, whereas the hydrogenation of 1-butene (red) is clearly preferred on Pd NPs of >4 nm. According to our computational modeling (see below), we attribute the latter to the smoother facets of the (111) type. The selectivity ratio (represented by the isomerization/hydrogenation ratio) thus ranges from 18 on smaller Pd NPs to 5 on the group of larger faceted Pd NPs, for which it is rather constant (Fig. 4b). The trend change in selectivity ratio with particle size is a direct result of the difference in slope for hydrogenation over particles <4 nm compared to larger particles, cf. Fig. 3d. The total average catalytic activity (1-butene consumption) for Pd NPs is very similar and close to 38 s^−1^; for details, see the SI. Modeling results that are used in a microkinetic model for various surfaces and subsequently combined to represent nanoparticles, see below, convincingly capture the trends of the TOF for hydrogenation (red inverted triangles), while the analogous isomerization results (green inverted triangles) scatter more. Despite the challenging nature of predicting TOFs and focusing the density functional theory (DFT) efforts solely on *trans*-2-butene, the modeling results represent quite well the overall experimental trends, including the selectivity ratio. As electronic structure and support effects can be excluded here, the different catalytic behavior has to originate from variations in the specific surface structure of the Pd NPs.

### Modeling

To rationalize the molecular origin of the particle size dependent selectivity ratio, we carried out density functional theory (DFT) calculations on slab models using the generalized gradient approximation (GGA) in the PBE variant^55^; for details, see Supplementary Notes 1 and 2 of the SI. The larger well-faceted Pd NPs were described by periodic models of the smooth surfaces Pd(111) and Pd(100), as well as Pd(211) representing edge sites, whereas for the surfaces of the smaller, rougher Pd NPs we chose periodic models of the surfaces Pd(211), Pd(110) and Pd(100) (Fig. 2); the (211) surface is stepped. Steps and edges increase the hydrocarbon binding energies and may serve as adsorption sites in experiments. Similar to our previous report^9^, the adsorption modes, free energies, and activation energies were calculated for all four types of surfaces; detailed results are provided as SI. The preferred reaction pathways were identified from these DFT results, Fig. 1.

**Fig. 5:**
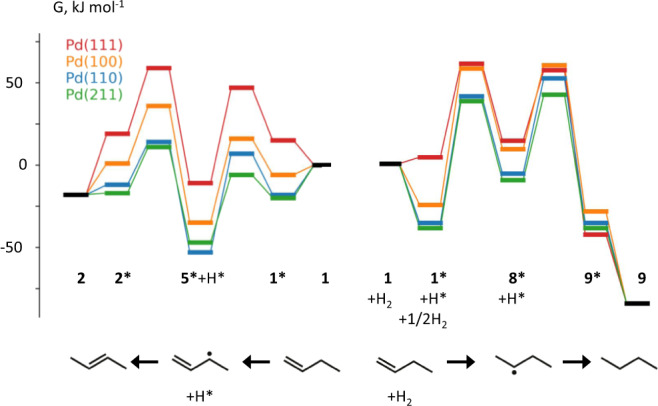
Calculated free energy profiles of the transformations of 1-butene, 1: isomerization (to the left) vs. hydrogenation (to the right). Lowest energy pathways only. For easy comparability of isomerization and hydrogenation, we start at the center with 1-butene **1** in the gas phase that adsorbs on Pd(111) (red), Pd(100) (orange), Pd(110) (blue), and Pd(211) (green). The *isomerization* of **1** to 2-butene, **2**, starts with a *dehydrogenation* of **1** to 1-buten-3-yl **5** and co-adsorbed H*. As reference of this transformation, we use the free energy value of 1-butene **1** in the gas phase at 373 K, 100 kPa; the partial pressure values were P_1-butene_ = 5 mbar and P_H2_ = 3.7 mbar with a standard surface concentration of θ° = 0.5. For the *hydrogenation* of **1** to butane **9**, we use 1-butene **1** and H_2_ as free energy reference. This transformation starts with a *hydrogenation* to 2-butyl **8**; see Fig. 1. In the final step of each reaction path, the product, 2-butene **2** or butane **9**, respectively, is desorbed into the gas phase. The free energy of 9* is shifted, by −40 kJ mol^−1^ from the DFT model results. This empirical correction reflects the challenging nature of modeling such processes by the DFT methodology applied; for details, see the text. This correction is inspired by experimental data on the typical entropy loss during the adsorption, from the gas, of saturated hydrocarbons on late transition metal surfaces.

The free energies in the following are calculated for 373 K and 100 kPa, mimicking experimental conditions. According to the DFT-derived free energy profiles of the lowest energy pathways for all surfaces, Fig. 5, the reaction barriers of 1-butene isomerization (1*→5*→2*) are lower than the barriers to hydrogenation (1*→8*→9*), thus rationalizing the experimentally determined preference for the isomerization. Adsorption energies from the gas phase range from slightly endothermic to somewhat exothermic for the overall hydrogenation, see also Supplementary Table S1 of the SI. For the hydrogenation path, hydrogen adsorption, formally 1/2 H_2_ at infinite separation from 1* and subsequent rearrangement, lowers the reaction energy of the initial step, Fig. 5. Furthermore, the isomerization shows a weak structure sensitivity (slightly lower barriers for the rougher surfaces), in agreement with the experimental results that isomerization is nearly independent of the particle size. In contrast, the hydrogenation of 1-butene exhibits a pronounced structure sensitivity, with Pd(111) clearly having the lowest barriers of all surfaces examined. In consequence, the hydrogenation of 1-butene is more efficient on Pd NPs larger than 4 nm, because these particles feature well-developed and abundant (111) facets^47^. The specific role of the (111) facets can be understood based on the particular reaction energy for the conversion of 1-butene 1* to butyl 8*, see below and Fig. 5. Note that the energy difference between 1* and 8* is calculated at ~50 kJ mol^−1^ for the surfaces (110), (100), and (211), but only 18 kJ mol^−1^ for (111). This difference in reaction energy translates to the barrier 1*→8*, being by at least 20 kJ mol^−1^ lower for (111) compared to other facets studied, Fig. 5 and Supplementary Table S2 of the SI. Already previous computational work determined a notably more favorable adsorption energy, 110 kJ mol^−1^, for butane at Pd(100) compared to Pd(111), 70 kJ mol^−1^ ^56^.

Turning to the DFT modeling results for the isomerization of 1- 1* to 2-butene 2*, we focus on the conversion to *trans*-butene, keeping in mind that *cis*-butene will be formed in analogous fashion^9^. On all Pd surfaces studied, the isomerization of 1* to 2* is found to proceed preferentially in a two-step mechanism, via initial dehydrogenation to 5* and subsequent hydrogenation to 2*, Fig. 1. The calculated barriers for the latter reactions on the closed-packed surface Pd(111) are rather high, with *G*_a_(1*→5*) = 32 kJ/mol and *G*_a_(5*→2*) = 70 kJ/mol, Fig. 5. The initial step is slightly exergonic, by −26 kJ/mol, while the hydrogenation to 2* is slightly endergonic, by 30 kJ/mol. Overall, the reaction energy of the isomerization over Pd(111) is 4 kJ/mol, i.e., the reaction is essentially thermoneutral. Note that on the surfaces Pd(100), Pd(110), and Pd(211) the free energy barriers of the dehydrogenation of 1* to 5* are reduced to 22 kJ/mol, 25 kJ/mol, and 14 kJ/mol, respectively. The associated reaction free energies Δ*G*_r_(1*→5*), −29 kJ/mol, −35 kJ/mol, and −27 kJ/mol, respectively, on these surfaces are also lower than the one calculated for Pd(111), thus promoting the dehydrogenation of 1* to 5*. The extremely low barriers of 1*→5* are followed by relatively high barriers for the formation of 2*, with Δ*G*_a_(5*→2*) calculated at 73 kJ/mol, 67 kJ/mol, and 58 kJ/mol for Pd(100), Pd(110) and Pd(211), respectively, Fig. 5. The somewhat lower barriers for the latter two surfaces, representing the small particles with rougher surfaces, may help to rationalize the slightly improved isomerization activity (TOF) of defective small Pd NPs compared to larger NPs.

An alternative path for the isomerization of 1-butene 1* on Pd catalysts proceeds via an initial hydrogenation reaction 1*→8* to form 8*, followed by the dehydrogenation 8*→2*; see 1 and Supplementary Fig. 1 of the SI. This pathway is known as the Horiuti-Polanyi mechanism^7,8^. Compared to the isomerization pathway via 5*, the path to 2* via 8* is calculated notably more favorable for Pd(111), but not for the other surfaces examined. The corresponding free energy barriers were determined at 57 kJ mol^−1^ and 56 kJ mol^−1^, whereas the free energy barrier for 5*→2* amounts to 70 kJ mol^−1^ for Pd(111). Barriers for 1*→8* on the surfaces Pd(100), Pd(110) and Pd(211) were calculated in the range 77–83 kJ/mol, somewhat higher than the route via 5*, Fig. 5. Moreover, the formation of 8* from 1* on Pd(100), Pd(110) and Pd(211) was determined highly endergonic, by ~50 kJ/mol. Overall, the Horiuti-Polanyi mechanism is less relevant for the catalyst under study, due to the endergonicity of 1*→8* for all surfaces studied, rendering the absolute barrier of 8*→2* higher than the barrier of 5*→2*.

Two alternative pathways also exist for the hydrogenation of 1-butene 1* to n-butane 9*, namely initial hydrogenation steps via either 1*→8* to 2-butyl 8* or 1*→7* to 1-butyl 7*, Fig. 1. On Pd(111), reaction 1*→8* has a lower barrier than 1*→7*, 57 kJ mol^−1^ vs 72 kJ mol^−1^, respectively. On Pd(110), the two alternative initial hydrogenation steps are calculated nearly degenerate, whereas for Pd(100) and Pd(211) 1*→7* is preferred by 7 kJ mol^−1^ and 9 kJ mol^−1^, respectively. As for the second hydrogenation step on these pathways, for all Pd surfaces explored, reaction 8*→9* exhibits a similar or slightly lower barrier than reaction 7*→9*, but is less exergonic than the latter. Unlike the other free energy values, that of butane 9* is a special case as the harmonic oscillator model severely overestimates the entropy loss upon adsorption. From experiments on butane over Pt(111)^57^, one derives a substantially lower entropy contribution to the desorption free energy, −34 kJ mol^−1^, for a monolayer coverage at 383 K. Based on this experimental result^57^, we applied a correction, −40 kJ mol^−1^, to the DFT free energy results of 9* for all Pd surfaces under study. At 373 K, adsorbed butane 9* will immediately desorb into the gas phase, not engaging in any reverse reaction. In any case, the free energy state of 9*, corrected with suitable experiments in mind, will affect neither the rate **1→9** nor the selectivity of **2** vs. **9** in the kinetic modeling. Note that the calculated free energy for the net gas phase reaction **1→9**, −85 kJ mol^−1^, agrees within 5 kJ mol^−1^ with the experimental value^58,59^.

In the following, we inspect the pathways 1*→5*→2* and 1*→8*→9* in more detail, as they provide a rather representative view on the barriers. Figure 5 summarizes the lowest free energy profiles of the isomerization, 1*→5*→2*, and the hydrogenation, 1*→8*→9*, as calculated for the Pd surfaces (111), (100), (110), and (211). The dehydrogenation of 1* to 5* is clearly preferred to the hydrogenation forming 8*. Therefore, the isomerization reaction is modeled as more favorable on all Pd particles. This observation is in close agreement with the high isomerization/hydrogenation ratios, irrespective of the Pd particle size. Interestingly, the lowest hydrogenation barriers are calculated for the close-packed surface Pd(111), while the surfaces Pd(110) and Pd(211) exhibit higher barriers, i.e., the hydrogenation seems to be rare on these corrugated surfaces. The lowest lying intermediate 5* does not bind as strongly on Pd(111) as an the other surfaces, concomitant with the relatively moderate barrier height for hydrogenation, overall yielding a higher propensity for butane formation than the other surfaces considered. At the other end of the selectivity ratio, Pd(211) binds intermediate 5* strongest, but also exhibits the lowest barrier to isomerization, while hydrogenation remains challenging.

**Fig. 6:**
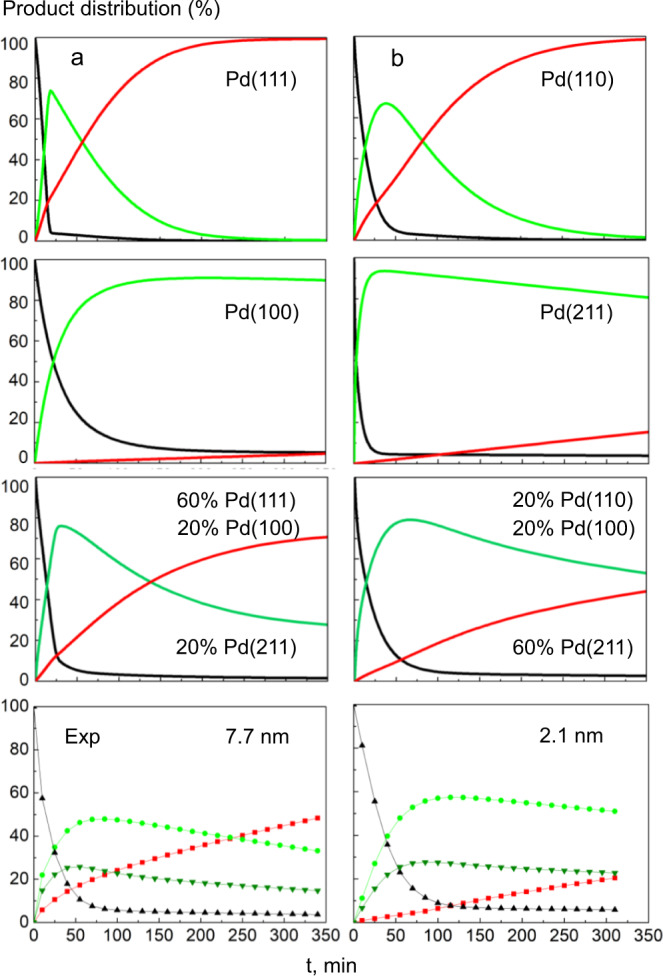
Simulated product distribution of 1-butene isomerization and hydrogenation as a function of the reaction time at 373 K. For **a** Pd(111), Pd(100), the mixture 60% Pd(111)/20% Pd(100)/20% Pd(211), and, at the bottom, the corresponding experimental results for average Pd NPs of 7.7 nm. **b** Analogous reactions simulated for Pd(110), Pd(211), the mixture 60% Pd(211)/20%Pd(110)/20% Pd(100), and the corresponding experimental results for average Pd NPs of 2.1 nm. Color coding: black—1-butene, red—n-butane, light green—*trans*-2-butene, dark green—*cis*-2-butene.

By appealing to the similarity between these stepped surfaces and the surface of rough particles, we thus are able to rationalize the experimental observations. Furthermore, the desorption of the fully hydrogenated product n-butane is an exergonic process for all Pd surfaces and it is expected to be most facile on Pd(111). For the isomerization product, at 373 K the desorption from Pd(111) is exergonic by −27 kJ mol^−1^, Supplementary Table 1. The desorption of *trans*-2-butene is exergonic on Pd(100), −9 kJ mol^−1^, and somewhat endergonic on the surfaces Pd(110) and Pd(211), by 5 kJ mol^−1^ and 15 kJ mol^−1^, respectively. Note that Fig. 5 depicts a 1/2 ML H coverage situation where the energy of 2* is calculated 10 kJ mol^−1^ more positive than at 1/16 ML H coverage^9^. Compared to 1-butene, the desorption of *trans*-2-butene is preferred by at least 10 kJ mol^−1^. A difference in DFT adsorption energies of 18 kJ mol^−1^ was previously determined on Pd(111)^60^. As expected, the desorption of butane from all surfaces is very exergonic. In a further modeling step, using the DFT results, we carried out microkinetic simulations for reaction conditions with 3.7 mbar H_2_ pressure to model the product distributions vs. time and compare them to the experiments, see Fig. 6 and Supplementary Notes 1 and 2 of the SI. This H_2_ pressure was shown to reproduce the experimental conversion rates for the single crystal surfaces Pd(111) and Pd(110) and thus was also applied herein^9^.

As demonstrated in Fig. 6a, the selectivity ratio and the rates on large faceted Pd NPs were well-mimicked by combining the results for (111), (100) and (211) surfaces in a 60:20:20% ratio (with (211) representing edge sites), according to typical distributions of surface atoms in the NP size range of 4–8 nm^24,38,47^. The closed packed Pd(111) surface allows hydrogenation, while Pd(100) and Pd(211) preferentially lead to isomerization Fig. 6b shows the corresponding simulation for small/rough Pd nanoparticles. Model results for Pd(211), Pd(110), and Pd(100) in a 60:20:20% relation again reproduce well the selectivity ratio and rates. The Pd(110) patches still allow hydrogenation, whereas the isomerization is mainly carried out on Pd(211) and Pd(100) patches. The hydrogenation reaction becomes notable only when the accumulated 2-butene **2** re-adsorbs on the surface. Given the various kinds of approximations and the typical error bar of ~10 kJ mol^−1^ for barrier predictions by DFT calculations^61^, the experimental catalytic performance is well reproduced (marked as inverted triangles in Fig. 4). The sensitivity to the quantitative combination of facets was probed using, among others, the alternative ratios 60/40 Pd(111)/Pd(100) for large particles and 70/30 Pd(211)/Pd(110) for small particles; see Supplementary Fig. 20 of the SI.

To verify experimentally that the hydrogenation of 1-butene mainly depends on the availability of (111) and (110) patches in the NP surface, an additional model catalyst was prepared that exhibits Pd NPs of 4.4 nm mean size (Fig. 2b and empty markers in Fig. 4). The average particle size of this sample is similar to that of the 5.3 nm Pd catalysts, but there were 3.6-times more nanoparticles/cm^2^ and 2.4-times more Pd surface atoms for the 4.4 nm catalyst. Accordingly, the overall conversion was larger, but the 4.4 nm NPs exhibited a similar selectivity ratio for n-butane as the 5.3 nm NPs, despite being prepared according to the procedure for the small rough NPs, i.e., at low substrate temperature. However, as shown by infrared spectra of adsorbed CO^45^, in this size range also particles with a “nominally rougher” surface develop better ordered facets. Accordingly, also the 4.4 nm Pd NPs offer low-index surface facets and thus exhibit a similar selectivity ratio for n-butane as the 5.3 nm NPs. The TOF values for hydrogenation are rather similar to those of larger particles, see the empty markers and arrows in Fig. 4a. In a temperature programmed reaction (TPR) study^23^ of *trans*-2-pentene hydrogenation in UHV, the activity was also assigned to (111) facets. On the other hand, for the 4.4 nm NPs, we did determine a yield of the isomerization product that is close to that of the “rougher” particles, making the 4.4 nm NPs a special “hybrid” case, see Fig. 2b. Accordingly, with our modeling effort, we were able to rationalize the particle size effect in atmospheric pressure 1-butene isomerization and hydrogenation on Pd/Al_2_O_3_ catalysts in an almost quantitative fashion.

## Discussion

We combined UHV-grown model catalysts (mean Pd particle size of 2–8 nm), atmospheric pressure batch reactor activity and selectivity studies, as well as DFT model results and microkinetic modeling for the Pd surfaces (111), (100), (110), and (211). All four surfaces exhibit low calculated barriers for isomerization, rationalizing the preference for isomerization and its weak particle size dependence. At variance, the higher barriers for hydrogenation show a strong structure sensitivity with the (111) surface (or such patches on NPs) exhibiting the lowest barriers.

Our modeling results suggest that, for all surfaces studied, the isomerization of 1-butene proceeds via an initial dehydrogenation of 1*, i.e., the removal of the allylic hydrogen, followed by a hydrogenation of the intermediate 5*. Our calculations also show that the first hydrogenation step, forming the intermediate 2-butyl 8*, governs the hydrogenation of 1* and they confirm that this alkyl intermediate is crucial on Pd(111), Pd(100), Pd(110) and Pd(211).

With these modeling results, one is able to rationalize in full the particle size dependence of 1-butene isomerization vs. hydrogenation. We conclude from this work that isomerization is achieved by small, rough NPs, that have no extended amounts of Pd(111) patches, whereas larger Pd nanoparticles (with their surface dominated by (111) facets) show higher rates for hydrogenation. Indeed, the size and amount of (111) surface patches, exhibited by larger particles, allow full hydrogenation of butene to butane. Microkinetic modeling demonstrated that isomerization usually precedes hydrogenation, with 2-butene being released to the gas-phase before it is re-adsorbed and converted to butane. This sequential reactivity could be further exploited for even improved selectivity.

In the current case, we were able to model indeed the properties of Pd nanoparticles via the properties of their individual surface facets and edge sites, which is the fundamental rationale of the surface-science single-crystal approach to heterogeneous catalysis. Clearly, this strategy does not apply to cases where other effects are active, may they be related to the electronic structure, metal/support interactions or inter-facet (intraparticle) interactions. Yet, the new insight presented may lead to an improved understanding and control of the selectivity of technologically relevant Pd catalysts.

## Methods

### Catalyst preparation

The experiments were carried out in an ultrahigh vacuum (UHV; base pressure ~ 1 × 10^−10^ mbar) surface analysis system combined with a UHV-high pressure reaction cell^47,52^. Pd/Al_2_O_3_ model catalysts, with a mean Pd particle size from 2 nm to 8 nm, were prepared by physical vapor deposition of Pd on a thin Al_2_O_3_ film (~0.5 nm) grown on clean NiAl(110)^38–40^. Samples were transferred under UHV to the reaction cell, where catalytic re-circulated batch measurements were performed at atmospheric pressure (P_1-butene_:5 mbar; P_H2_:10 mbar; Ar added to 1 bar) at 373 K, with reaction products quantified by on-line gas chromatography (GC).

### Computational details

We carried out plane-wave based DFT calculations using the Vienna ab-initio simulation package (VASP)^62,63^. We applied the generalized gradient approximation (GGA) as proposed by Perdew, Burke, and Ernzerhof (PBE) as exchange-correlation functional^55,64^. We used the projector-augmented wave (PAW) method to describe ionic cores^65,66^. The cutoff energy for the plane-wave basis set was 400 eV. A first-order Methfessel-Paxton smearing with a width of 0.1 eV was applied^67^. A Monkhorst-Pack mesh of 5 × 5 × 1 *k* points was used to sample the Brillouin zone. Geometries were considered converged when the force on each atom was below 2 × 10^−4^ eV pm^−1^.

More details on catalyst preparation, characterization methods, catalytic data, and modeling are described in the Supplementary Information. This material is available free of charge via the Internet.

### Reporting summary

Further information on research design is available in the Nature Research Reporting Summary linked to this article.

## Supplementary information

Supplementary Information

Reporting Summary

## Data Availability

The Cartesian coordinates of intermediates and transition states generated in this study have been deposited at Gitlab under https://gitlab.com/agenest/pd-particles. Any other data that support the findings of this study are available from the corresponding author upon reasonable request.
