# Supplementary material for: The origin of the particle-size-dependent selectivity in 1-butene isomerization and hydrogenation on Pd/Al2O3 catalysts
Source: Nat Commun. 2021 Oct 20;12:6098. doi: 10.1038/s41467-021-26411-8 (PMC8528898; doi:10.1038/s41467-021-26411-8)
Supplement: Supplementary file 1 — Supplementary Information [file 41467_2021_26411_MOESM1_ESM.pdf]

The origin of the particle-size-dependent selectivity in 1-butene isomerization and hydrogenation on Pd/Al_2O_3 catalysts

Alexander Genest,[1,2,≠] Joaquín Silvestre-Albero,[1,3,≠] Wen-Qing Li,[2,≠]
Notker Rösch,[1,4] and Günther Rupprechter[1,*]

[1] *Institute of Materials Chemistry, Technische Universität Wien, Getreidemarkt 9/BC, A-1060 Vienna (Austria)*
[2] *Institute of High Performance Computing, Agency for Science, Technology and Research, 1 Fusionopolis Way, #16-16 Connexis, Singapore 138632 (Singapore)*
[3] *Permanent address: Laboratorio de Materiales Avanzados, Universidad de Alicante, Ap. 99, E-03080 Alicante (Spain)*
[4] *Department Chemie and Catalysis Research Center, Technische Universität München, D-85747 Garching (Germany)*

[≠] *These authors contributed equally*

Supplementary Information

Supplementary Note 1: DFT modeling

Plane-wave-based DFT calculations were carried out on periodic slab models using the Vienna Ab initio Simulation Package (VASP).[1,2] As exchange-correlation functional we chose the Perdew-Burke-Ernzerhof (PBE) version of the generalized gradient approximation (GGA).[3,4] To assess the range of adsorption energies, we also applied the BEEF-vdw functional,[5] which includes by default an ensemble of non-selfconsistently evaluated density functionals, thus providing an error estimate via the standard deviation of these values. For modeling the ionic cores we invoked the projector-augmented wave (PAW) method.[6,7] We set the cut-off energy for the plane-wave basis set to 400 eV. Furthermore, we used the first-order Methfessel-Paxton "smearing" with a width of 0.1 eV;[8] all energies were extrapolated to 0 K. The Brillouin zone of the various slab models (see below) was sampled with a Monkhorst-Pack mesh of 5×5×1 k-points. The self-consistent field procedure was considered converged when the change in the total energy was at most 1×10^{-6} eV. All geometries were optimized until the Hellman-Feynman force on each atom was less than 2×10^{-4} eV pm^{-1}. We determined approximate structures of the transition states with the climbing-image nudged elastic band method (CI-NEB).[9] Subsequently we refined these structures with the dimer method.[10] The PBE calculated lattice constant of bulk Pd is 394 pm, i.e., 1.5% longer than the corresponding experimental value, 388 pm.[11]

We examined the reaction mechanisms of 1-butene transformations using slab models of the four Pd surfaces (111), (100), (110), and (211). A 4×4 surface was chosen for the Pd(111) surface and a 5×3 surface for Pd(100), each both comprising five atomic layers. These models were taken to represent (111) and (100) facets of large Pd NPs. Small Pd NPs, with a high density of step defects at the surface, were represented by the stepped surfaces Pd(211) and Pd(110), using 4×3 surface unit cells. These latter models formally comprise 16 and 10 layers of Pd atoms, respectively, and thus may be taken as equivalent to 5 atomic layers of Pd(111). In all models, the repeated slabs in the *c* direction of the supercell were separated by a gap of at least 1.5 nm. The "top" three atomic Pd layer equivalents of all models were fully relaxed during structure optimization while the "bottom" two Pd layer equivalents were kept fixed at the optimized bulk structure. The Pd atoms of the "top" most layer equivalent were counted to evaluate the coverage, 1/16 ML for Pd(111), 1/18 ML for Pd(100), 1/12 ML as well as for Pd(110) and Pd(211).

Previously, we modeled isomerization and hydrogenation reactions of 1-butene at the single crystal surfaces Pd(111) and Pd(110)[12] where the Cartesian coordinates of all pertinent the stationary structures on these latter two surfaces have been provided. To further understand the

reactions occurring on Pd(100) and Pd(211) surfaces, we also studied the corresponding reaction network, cf. Supplementary Figure 1. The first step in the isomerization process, 1-butene **1*** to 2-butene **2***, is a dehydrogenation to 1-buten-3-yl **5*** intermediate, followed by a hydrogenation step to 2-butene **2***, see reactions R4 and R5, Supplementary Figure 1. The so-called Horiuti-Polanyi mechanism[13] offers an alternative path to the 2-butene intermediate **2*** via a hydrogenation step to 2-butyl **8***, followed by a dehydrogenation, see reactions R6 and R7.

In the full hydrogenation to n-butane **9***, the intermediates 2-butyl **8*** or 1-butyl **7*** are involved, see reactions R6, R8, R9, and R10, Supplementary Figure 1. Other dehydrogenation reactions R1 to R3, leading to intermediates **3***, **4***, and **6***, can be ruled out due in view of higher energy barriers, Supplementary Figure 2. The hydrogen atoms that participate in the isomerization and hydrogenation of 1-butene transformations were taken to be co-adsorbed in the immediate vicinity of the hydrocarbon, see Supplementary Figures 2, 3. When required for a reactions step, we assumed all associated hydrogen diffusion events to be facile. Cartesian coordinates of all pertinent stationary structures, determined for the surfaces Pd(100) and Pd(211) are provided as additional Supplementary Information.

We estimated Gibbs free energies of adsorption by adjusting pertinent electronic energies for the zero-point energy correction and the entropy correction at 373 K, approximated by the harmonic oscillator model. Hereby we invoked the vibrational degrees of freedom of a complex that involves the adsorbate and the metal centers directly involved in the adsorption as well as the direct metal neighbors of the latter centers. Surface species were assumed not to engage in frustrated rotations or translations. The standard pressure of these free energies is 1 bar for species in the gas phase, while for adsorbed molecules, treated as lattice gas, the standard surface concentration is 0.5.[14,15] For species in the gas phase, we also considered the rotational degrees of freedom. For molecular species in the gas phase, these corrections were determined using the program Gaussian 09,[16] at the analogous level of theory using basis sets of the double zeta type. Modes with vibrational frequencies below 100 cm^{-1} or imaginary frequencies (of at most 50 cm^{-1}) were assigned a frequency of 100 cm^{-1} for evaluating the entropy term.

Adsorption energies ΔG_{ads} and reaction energies ΔG_{r}, were calculated according to

$$\Delta G_{\mathrm{ads}} = G_{\mathrm{ads}} - (G_{\mathrm{gas}} + G_{\mathrm{slab}}) \tag{1}$$

$$\Delta G_{\mathrm{r}} = G_{\mathrm{pro}} - G_{\mathrm{rea}} \tag{2}$$

where G_{ads} are free energy values of adsorption complexes, and the term $G_{\mathrm{gas}} + G_{\mathrm{slab}}$ is the sum of total free energy values of the molecule in the gas phase and the bare Pd surface, respectively,

Supplementary Table 1. The terms G_{pro} are the free energy values of the adsorption complexes of the product and the reactant, respectively. Relative activation barriers, ΔG_a, are referenced to the local minimum immediately preceding the transition state.

To control the computational effort, we estimated the influence of H coverage at the surface on the Gibbs free energies of elementary reaction steps, see Supplementary Tables 2, 3. We identified the most important reactions of the reaction network applying a sensitivity analysis.[17] Barriers and reaction energies that lead to isomerization or hydrogenation (R4, R5, R6, R7, R8, R9, and R10, Supplementary Table 3), were then evaluated for Pd(111) using a hydrogen coverage of 0.5 ML. The energy differences between the models without extra H or with a 0.5 ML hydrogen coverage were then also applied to the corresponding energies of the remaining surfaces Pd(110), Pd(100), and Pd(211), Supplementary Table 2. One needs to correct both reaction energies, by 10 kJ mol^{-1} for **1→5→2** as well as 21 kJ mol^{-1} for **1→8→9**. Ultimately, this accumulated correction was removed at the stage of the stable species **2** and **9**, respectively; see Supplementary Table 3.

Supplementary Note 2: Microkinetic Modeling

In the model developed, we evaluated the microkinetics between adsorption/desorption events A/D and reactions R occurring on the Pd surfaces that represent the network of elementary surface reactions shown in Supplementary Figure 1; see also Supplementary Tables 1, 2. With the help of the software library BzzMath,[18] we solved a system of rate equations to quantify the concentrations of reactants and products, and the surface coverages θ of the adsorbed species. For 1-butene hydrogenation at 373 K on Pd surfaces microkinetic modeling uses partial pressures of 5.0 mbar for 1-butene and 3.7 mbar for H_2. We used the following site densities for the various surfaces: 2.13×10^{-5} mol m^{-2} for Pd(100), 1.56×10^{-5} mol m^{-2} for Pd(110), 2.58×10^{-5} mol m^{-2} for Pd(111), 1.89×10^{-5} mol m^{-2} for Pd(211).

Evaluation of rate constants

We calculated the rate constant k_i(fwd) of the i-th forward reaction step as

$$k_i(fwd) = A\ exp\left(-G_{a(i)}/RT\right) = (k_B T)/h\ exp\left(-G_{a(i)}/RT\right) \quad (3)$$

Here $G_{a(i)}$ is the standard state Gibbs activation free energy of reaction i (kJ mol^{-1}) containing the terms as specified in the previous section, A is the pre-exponential factor, k_B is the Boltzmann constant (J K^{-1}), h is Planck's constant (J s), T is the temperature (K), and R is the gas constant (kJ K^{-1} mol^{-1}). The Gibbs free energies of the initial states and the corresponding transition state structures were calculated in advance, see Supplementary Note 1. Following our previous work,[12] we evaluated rates using these rate constants times surface concentrations times the actual surface area of our catalyst in experiment.

Assuming the adsorption reactions A1, A2, as well as those reverse to D1, and D2, Supplementary Table 1, to proceed without activation, i.e., to be reversible, one may describe their rate by collision theory as[19]

$$k_{ads} = S_0 \frac{A_{site} 10^5}{\sqrt{2\pi m k_B T}} \quad (4)$$

Here, the mass of the molecule m is in kg, the Boltzmann constant k_B in J K^{-1}, and the temperature T in K. A_{site} corresponds to the surface area per adsorption site (m^{-2}), calculated in our study as A/N, i.e., the surface area A divided by the total number of surface atoms N, both determined from experiment, see Supplementary Note 3. The factor 10^5 in the numerator converts the units of the pressure for adsorption from Pascal to bar. The initial sticking coefficient S_0 of a molecule in the gas phase is set to 1 for 1-butene, 2-butene, and n-butane, and to 0.1 for H_2.[20,21]

For thermodynamic consistency of the constructed model, we determined the rate constant of the reverse reaction of an elementary step i (surface reactions and adsorption reactions) from the pertinent equilibrium constant $K(i)_{eq}$:

$$k_{i_{rev}} = k_{i_{fwd}}/K(i)_{eq} \quad (5)$$

where $K(i)_{eq}$ is derived from the reaction free energy of the elementary step under consideration, Supplementary Table 2:

$$K(i)_{eq} = exp\left(-G_{r(i)}/RT\right) \quad (6)$$

Strategy for determining the amount of blocked sites

According to scanning tunneling microscopy (STM) characterization,[22] 2-butene blocks N_{bs} = 2–3 metal centers, which we use as basic unit of "site" at the surface Pd(110). A similar value of N_{bs} was also deduced in our previously calculated structure of 2-butene on Pd(110).[12] For the surfaces Pd(111), Pd(100) and Pd(211), we were unable to locate pertinent experimental information. To treat the surfaces studied, Pd(110), Pd(111), Pd(100), and Pd(211), on equal footing, we scaled the value of N_{bs} for Pd(110) for the other surfaces with the surface density ρ of the Pd atoms in the first layer equivalent, Supplementary Table 4. For example, taking N_{bs} = 2 blocked sites for Pd(110), we obtain 2 sites × ρ[Pd(111)] / ρ[Pd(110)] = 3.27 blocked sites for Pd(111); we round this value to N_{bs} = 3, Supplementary Table 4. The site densities are in the order, Pd(111) > Pd(100) > Pd(211) > Pd(110). As we determine 2 or 3 blocked sites on Pd(110), we arrive at ranges of N_{bs} for the other surfaces. This scaling procedure results in 3–5 blocked sites for Pd(111), 2–5 for Pd(100), and 2–4 for Pd(211), Supplementary Table 4.

We probed the influence of the number of blocked sites by varying their number for each surface.[12] Ultimately, we used 4 blocked sites for Pd(111), 2 for Pd(100), 2 for Pd(110) and 3 for Pd(211) to describe the space requirement of adsorbed 2-butene. We kept the number of blocked sites mostly constant throughout the various reaction pathways. For intermediate **5*** on corrugated surfaces, we increased the N_{sb} value by one with respect to other species, Supplementary Table 5. Conversely, we used N_{sb} values decreased by one on all surfaces for the nearly saturated intermediates **7***, **8***, and **9*** which are only bound through one C-metal bond.

Micro-kinetic modeling, adapted rate constants of the desorption reactions

Micro-kinetic modeling was carried out using rate constants derived from Gibbs free reaction energies and activation energies as just described, Supplementary Table 2. Supplementary Figures 4c and 4d show the resulting calculated product distributions of 1-butene transformation on the surfaces Pd(111) and Pd(110). We note some deviation between the predictions and the experimental measurements, Supplementary Figures 4a and 4b. The conversion of 1-butene over time has a similar trend as in the measurements, while the model yields a notably slower hydrogenation to butane (Supplementary Figure 5, blue lines) than found in the experiments, leading to a high selectivity for trans-2-butene.

As in our previous work, we carried out a sensitivity analysis[17] regarding the formation of n-butane, to elucidate the influence of individual elementary reactions within the network.[12] To this

end, the rate constants k_i were perturbed, one at a time, increasing their values by 10%. At the same time, the equilibrium constant K_i^{eq} of reaction *i*, remained unchanged to maintain the thermodynamic equilibrium condition. Hence, for each elementary reaction step *i* of the reaction network, a calculation with the perturbed parameter k_i was carried out to determine the variation of the output quantity X_k. In this work, we chose $X_0 = 9\%$ as output quantity, i.e., 9% of the product n-butane was formed. For a rate constant k_i, the normalized sensitivity coefficient NSC_i is expressed as:

$$NSC_i = d \ln X / d \ln k_i \approx (k_i/\Delta k_i)\,(\Delta X/X) = [k_{i,0}/(k_i - k_{i,0})][(X_i - X_0)/X_0] \qquad (7)$$

The value (positive or negative) of NSC_i indicates the magnitude of the influence of reaction *i* on the formation of the products.

Supplementary Figure 6 shows the impact on the formation rate of the hydrogenation product, n-butane. Accordingly, the adsorption A1 of 1-butene, the hydrogenation R6 of 1-butene, and the hydrogenation R8 of 2-butyl **8*** have the highest positive sensitivity coefficient for reactions on Pd(111) and Pd(110), whereas we obtained large negative coefficients NSC_i for the desorption D1 of trans-2-butene.

As we identified the fast desorption reaction to be responsible for the rather low hydrogenation activity, we subsequently explored likely causes of this observation. The desorption is controlled by the desorption energy of an adsorbate. i.e., the negative of the corresponding adsorption energy on a metal surface. The calculated desorption energy, in turn, is affected by the chosen exchange-correlation functional and the associated gain of entropy. While calculations using the PBE functional have their advantages in describing reactions, they have been shown to underestimate desorption energies, on the example of hydrocarbons at Pt(111).[23] Using the BEEF-vdW functional, which had been reported to yield more realistic adsorption/desorption energeties,[23] we re-evaluated the desorption energies of the structures obtained with the PBE approximation, Supplementary Table 1. With the BEEF-vdW functional, we indeed identified rather similar adsorption energies of 1-butene, between -77 kJ mol^{-1} and -82 kJ mol^{-1} for Pd(111), Pd(110), and Pd(100), Supplementary Table 1. On Pd(211), we determined a somewhat more favorable adsorption energy, -97 kJ mol^{-1}, Supplementary Table 1. In addition, we note that the standard deviations of adsorption energies reported with the BEEF-vdW functional are between 23 kJ mol^{-1} and 30 kJ mol^{-1}, indicating a notable uncertainty. For 2-butene the resulting adsorption energies are 3 kJ mol^{-1} to 10 kJ mol^{-1} lower than for 1-butene, but similar in trend. For Pd(111) and Pd(110), we calculated about -77 kJ mol^{-1}, and -68 kJ mol^{-1} for Pd(100); Pd(211) again yields the highest

value in absolute terms, -87 kJ mol^{-1}. Also, the error ranges remain comparable to those obtained for 1-butene.

Inspired by the BEEF-vdW results, we revisited our microkinetic model and increased the desorption energies for Pd(111), Pd(110), and Pd(100) by 23 kJ mol^{-1}, which renders the surfaces Pd(111) and Pd(110) comparable to the experiment, Supplementary Figures 6, 7. To keep this correction as consistent as possible, we resorted to applying the same correction in the case of Pd(100), but used a smaller increment, 18 kJ mol^{-1}, to the desorption energies on Pd(211) as the PBE values of both adsorbates, 1-butene and 2-butene, were already larger, Supplementary Table 6. These increased desorption energies reflect the role of stronger binding edges available in the experimental NPs, where species may first adsorb, then undergo an initial reaction step, and subsequently the resulting intermediates may engage in diffusion at the surface. This also would result in rather uniform adsorption kinetics for all inspected surfaces, as such edges are available in general.

Finally, we consider the effect of frustrated rotations and translations on the calculated free energies of adsorption/desorption to be covered by the corrections just described, but refrained from a more detailed specification in our model in view of uncertainties in the model and the experimental values.[24]

Strategy for mimicking Pd NPs by mixing results from several Pd surfaces

Pd nanocrystals with a mean particle size below 4 nm, typically showing round/irregular outlines in STM, have rough surfaces, with many defects including steps. This has been demonstrated by infrared spectra of adsorbed CO which show strong or even dominating on-top peaks, see Supplementary Note 3. In contrast, Pd nanocrystals of 4 nm and larger mean size exhibit well-developed structures of truncated cuboctahedra (hexagonal outlines in STM), with about 50–65% surface atoms in (111) facets, 10–20% in (100) facets and a remainder of edges and perimeter sites.

To rationalize the molecular origin of the particle size dependent selectivity of 1-butene hydrogenation, we resorted to combining the results of microkinetic modeling of various crystalline surfaces. To model small rough Pd NPs with diameters of about 2–4 nm, combining results for surface facets of stepped Pd(211), (110) and (100) appears suitable. It seems appropriate to model large well-facetted Pd NPs of about 5–8 nm by combining results for the smooth surfaces Pd(111), (100), and (211). Therefore, micro-kinetic simulations were applied to simulate the

experimental measurements (product distribution in time, see Experimental Section). Based on the calculations for the four single crystal terminations examined, the catalytic properties of small and large Pd NPs were mimicked by combining the properties of the surfaces (211)+(110)+(100) and (111)+(100)+(211), respectively. In both cases, we assumed a ratio of 60:20:20%, see Supplementary Figures 8 and 9, respectively. Supplementary Figure 11 depicts an alternative mixing ratio.

Supplementary Note 3: Experimental setup and catalyst preparation

The experiments were carried out in an ultrahigh vacuum (UHV) surface analysis system combined with a UHV-high pressure reaction cell.[25,26] For sample characterization, the UHV section (base pressure ~ 1×10^{-10} mbar) was equipped with low energy electron diffraction (LEED), temperature programmed desorption (TPD), and X-ray photoelectron spectroscopy (XPS).[27-29]

Pd/Al_2O_3/NiAl(110) model catalysts were prepared by growing a thin ordered Al_2O_3 film (thickness ~0.5 nm) on clean NiAl(110) by two cycles of oxidation in 10^{-6} mbar O_2 at 543 K (20 min) and subsequent annealing to 1100 K in UHV (3-5 min), followed by Pd (purity ≥ 99.99%) electron beam evaporation at 90 K or 300 K substrate temperature.[25-29] Pd nanoparticles with a mean particle size from 2 nm to 8 nm were prepared by varying the substrate temperature and the Pd amount deposited.

As revealed by room temperature STM, Supplementary Figure 13, Pd nanoparticles larger than 4 nm (grown at 300 K) typically have the shape of truncated cubo-octahedra with distinct smooth (111) and (100) facets, whereas smaller particles (grown at 90 K) appear rather rounded/irregular (i.e., the facets are rougher with more defects).[27,28,32-34] All catalysts prepared exhibited a rather narrow particle size distribution (mean particle size ±~1 nm). The STM-derived surface structure has been confirmed by vibrational spectroscopy using CO as probe molecule.[25-27,33-36]

Supplementary Table 7 collects the structural properties of the various Pd/Al_2O_3/NiAl(110) catalysts. After preparation, all samples were annealed to 373 K to exclude thermally-induced structure changes during reaction. Independent STM studies indicated that NP sintering only occurred above 773 K.[39]

Catalytic batch reactor measurements and activity/selectivity

The clean samples were transferred under UHV to the reaction cell, where catalytic measurements were performed at atmospheric pressure. The model catalysts were exposed to the reaction mixture (high-purity $P_{1\text{-butene}}$: 5 mbar; P_{H2}: 10 mbar; Ar added to 1 bar) at a temperature of 373 K. Kinetic measurements were carried out in a batch mode with the gas recirculating over the catalyst by a metal bellows pump (reactor volume exchanged 5 times per minute). The reaction products were analyzed by on-line gas chromatography (GC), using a HP-PLOT/Al_2O_3 column (50m×0.53mm) capillary column and a flame ionization detector FID.[30] Retention times and sensitivity factors for the reactant and products were calibrated using different gas mixtures. Employing Al_2O_3 films on NiAl(110) as "inert" catalysts, the absence of background (wall) and support reactions was confirmed.

To avoid the formation of β-palladium hydride, the pressure P_{H2} of H_2 was kept well below the required threshold pressure (about 20 and 200 mbar at room temperature and 373 K, respectively).[40] Consequently, the catalytic reactions were started on well-defined clean surfaces of the Pd NPs. Given the "mild" reaction conditions, major structural/compositional changes of the Pd nanoparticles can be largely excluded. When the reaction was stopped and the reaction cell recharged with the original reaction mixture, hardly any differences in the GC reaction profiles were observed.

In any case, we focused on the initial activity and selectivity of the model catalysts i.e., after 10 min reaction time, when both the cleanliness and structure were hardly affected. Supplementary Table 8 reports the turnover frequencies of the various catalysts for 1-butene isomerization and hydrogenation, the selectivity (the iso/hydro ratio), and the trans-2-butene/cis-2-butene ratio.

Supplementary Table 1. Adsorption free energies G_{ads} calculated at 373 K of the reactant 1-butene **1*** and the products of its *isomerization*, 2-butene **2*,** and *hydrogenation*, butane **9*** on various Pd surfaces. Adsorption energies E_{ads} obtained with the BEEF-vdW functional[a] in single-point fashion, including the associated standard deviation, are reported for comparison. Values including correction (corr) as used in the microkinetic model. All values in kJ mol^{-1}.

Label	Adsorbate	Energy	Functional	Pd(111)	Pd(110)	Pd(100)	Pd(211)
A1	1-butene	G_{ads}	PBE	15	-18	-6	-20
			corr	-8	-41	-29	-38
		E_{ads}	PBE	-61	-92	-80	-96
			BEEF-vdW[b]	-82±30	-81±23	-77±26	-97±30
A2	1/2 H_2	G_{ads}	PBE	-30	-26	-24	-33
D1	2-butene	G_{ads}	PBE	27	-5	9	-7
			corr	4	-28	-14	-25
		E_{ads}	PBE	-51	-81	-68	-85
			BEEF-vdW[b]	-76±32	-78±24	-68±27	-87±28
D2	butane	G_{ads}	PBE	82	89	96	86
			corr	59	66	73	68
		E_{ads}	PBE	-7	-17	-11	-14

[a] Ref. 5. [b] Calculated in single-point fashion for structures determined at the PBE level.

Supplementary Table 2. Gibbs free reaction energies G_r and activation energies G_a (kJ mol^{-1}) on four Pd surfaces, calculated for the isomerization and the hydrogenation pathways of 1-butene at 0.5 ML H coverage. Analogous activation energies E_a and reaction energies E_r. See Supplementary Figure 2 for the labels of the species.

		Pd(111)				Pd(110)				Pd(100)				Pd(211)			
	Steps	G_a	G_r	E_a	E_r	G_a	G_r	E_a	E_r	G_a	G_r	E_a	E_r	G_a	G_r	E_a	E_r
	**1* + * ↔ 1* + H***		-11		-35		-18		-45		-19		-47		-19		-48
	**7* + * ↔ 7* + H***		-10		-35		-19		-44		-21		-48		-21		-43
	**8* + * ↔ 8* + H***		-8		-34		-21		-47		-16		-46		-21		-44
R1	**1* ↔ 3* + H**	93	3	-107	-16	76	11	-87	-22	87	-5	-101	-9	62	11	-71	-18
R2	**1* ↔ 4* + H**	89	-9	-110	-2	70	6	-85	-22	75	-11	-94	-3	62	4	-80	-24
R3	**1* ↔ 6* + H**	75	42	-85	-47	61	16	-78	-30	60	9	-68	-24	80	30	-93	-45
R4	**1* ↔ 5* + H**	32	-26	-43	-11	25	-35	-36	-17	22	-29	-31	-14	14	-27	-22	-2
R5	**5* + H ↔ 2***	70	30	-69	-17	67	40	-62	-24	73	36	-70	-22	58	30	-42	-5
R6	**1* + H ↔ 8***	57	18	-60	-8	77	51	-75	-36	83	50	-86	-40	77	50	-78	-39
R7	**8* ↔ 2* + H**	56	-14	-69	-3	43	-49	-56	-32	30	-52	-41	-29	45	-44	-54	-29
R8	**8* + H ↔ 9***	43	-17	-49	-29	58	10	-57	-13	51	2	-46	-18	52	11	-48	-9
R9	**1* + H ↔ 7***	72	34	-77	-22	81	74	-82	-54	76	73	-77	-53	68	76	-71	-59
R10	**7* + H ↔ 9***	43	-34	-47	-43	66	-16	-65	-35	60	-20	-59	-33	57	-16	-58	-30

Supplementary Table 3. Reaction free energies G_r and free energy barriers G_a of various reaction steps[a] on Pd(111), calculated at two values of hydrogen coverage, 1/16 ML and 0.5 ML. Energies in kJ mol^{-1}.

		G_a		Δ^b	G_r		Δ^b
	H coverage	1/16 ML	0.5 ML		1/16 ML	0.5 ML	
R4	**1* ↔ 5* + H**	32	32	0	-38	-26	12
R5	**5* + H ↔2***	69	70	1	32	30	-2
R6	**1* + H ↔ 8***	63	57	-7	10	18	8
R7	**8* ↔ 2* + H**	55	56	1	-13	-14	-1
R8	**8* + H ↔ 9***	55	43	-12	-30	-17	13
R9	**1* + H ↔ 7***	74	72	-2	11	34	24
R10	**7* + H ↔ 9***	44	43	0	-29	-34	-5

[a] For the various reaction steps, see Supplementary Figure 2. [b] Δ – Energy difference between the two coverages. The deviations of the free energy values of the various reaction steps are calculated for Pd(111) and then used to estimate the activation energies and reaction energies at 0.5 ML H coverage on Pd(110), Pd(100), and Pd(211), see Supplementary Table 2.

Supplementary Table 4. Site density ρ, i.e., accessible metal centers per area,[a] (in 10^{-5} m^{-2} mol^{-1}) in our computational surface structures; experimental values from the main manuscript. N_{bs}(min) – number of blocked sites scaled by the site density of the surface and rounded down, when N_{bs} = 2 is chosen for Pd(110); N_{bs}(max) –number of blocked sites scaled by site density of the surface and rounded up, when N_{bs} = 3 is selected for Pd(110); N_u – number of blocked sites used.

Surface	ρ		N_{bs}(min)	N_{bs}(max)	N_u
	Theo	Exp			
(111)	2.47	2.58	3	5	4
(110)	1.51	1.56	2	3	2
(100)	2.13		2	5	2
(211)	1.89		2	4	3

[a] Projection of the unit cell onto the x,y-plane.

Supplementary Table 5. Blocked sites N_u used for all pertinent intermediates (Int.) on the four surfaces Pd(111), Pd(110), Pd(100), and Pd(211). See Supplementary Figure 2 for the labels of the species.

Int.	Pd(111)	Pd(110)	Pd(100)	Pd(211)
**1***	4	2	2	3
**2***	4	2	2	3
**3***	4	2	2	3
**4***	4	2	2	3
**5***	4	3	2	4
**6***	4	2	2	3
**7***	3	1	1	2
**8***	3	1	1	2
**9***	3	1	1	2

Supplementary Table 6. Adjustments Δ, kJ mol^{-1}, of the free energy G_{ads} of the elementary desorption energy values given in Supplementary Table 1. See the discussion in Supplementary Note 2.

	Δ
Pd(111)	23
Pd(110)	23
Pd(100)	23
Pd(211)	18

Supplementary Table 7. Structural properties of various UHV-grown $Pd/Al_2O_3/NiAl(110)$ model catalysts. Given are the substrate temperature during evaporation Tmp, the Pd nominal thickness th, the island density Isl Dens, the mean particle diameter <d>, the number of Pd surface atoms per nanoparticle $Pd_{s,NP}$, the number of Pd atoms per nanoparticle Pd_{NP}, the dispersion Disp of Pd, the total number Pd nanoparticles in the sample $Pd_{NP,\ tot}$, and the total number of Pd surface atoms in the sample $Pd_{s,\ tot}$.

Tmp K	th[a] nm	Isl Dens[b] 10^{12} cm^{-2}	<d>[c] nm	$Pd_{s,NP}$	Pd_{NP}	Disp %	$Pd_{NP,\ tot}$[d] 10^{12}	$Pd_{s,\ tot}$[d] 10^{14}
90	0.2	8.7	2.1	96	157	61	6.79	6.5
	0.4	6.3	2.8	189	440	43	4.91	9.3
	0.6	4.7	3.6	300	882	34	3.67	11
	0.8	3.6	4.4	427	1472	29	2.81	12
303	0.4	1	5.3	638	2658	24	0.78	5.0
	0.6	1	6.1	846	4029	21	0.78	6.6
	1.2	1	7.7	1277	7981	16	0.78	10

[a] Values measured by a quartz microbalance thickness monitor.
[b] Values obtained by *in situ* STM measurements.[27,41]
[c] Values calculated for hemispherical nanoparticles based on the deposited Pd amount and the island density measured by STM (due to tip-convolution effects, NPs appear larger in STM images).
[d] For a sample area of 0.78 cm^2.

Supplementary Table 8. Turnover frequency TOF (s^{-1}) after 10 min from experimental measurements and modeling estimates (373 K). Hyd − hydrogenation of n-butane, t-iso − isomerization of trans-2-butene, ratio – the ratio isomerization vs hydrogenation. For modeling nanoparticles (< 4.0 nm), products from surfaces Pd(211), Pd(110), and Pd(100), mixed in a fixed ratio 60:20:20. For modeling nanoparticles (> 4.0 nm), products from the surfaces Pd(111), Pd(100), and Pd(211), mixed in a fixed ratio of 60:20:20.

	Expt.			Theo.		
Size of NP	t-iso	Hyd	ratio	t-iso	Hyd	ratio
2.1	21.7	1.9	18.2	33.8	3.1	10.9
2.8	21.0	2.2	14.8	27.8	3.0	9.3
3.6	21.8	3.1	10.9	25.3	3.0	8.6
4.4	22.3	6.4	5.2	32.7	5.9	5.6
5.3	18.8	6.3	5.0	34.1	5.6	6.0
6.1	17.7	6.5	4.5	33.2	5.8	5.8
7.7	18.9	6.5	4.7	32.8	5.9	5.6

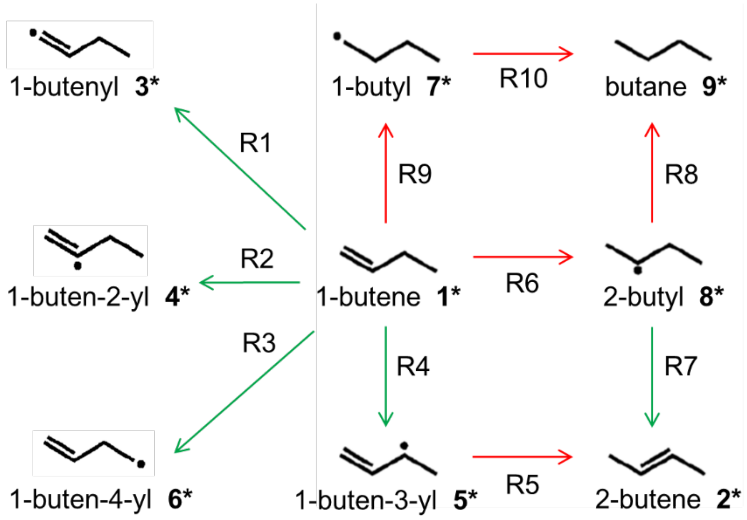

Supplementary Figure 1. Reaction network of the transformations of 1-butene on Pd. The labels for the reactions and various species are used throughout the main manuscript and the Supplementary Information. Hydrogenation reactions are designated via red arrows, dehydrogenation as green arrows.

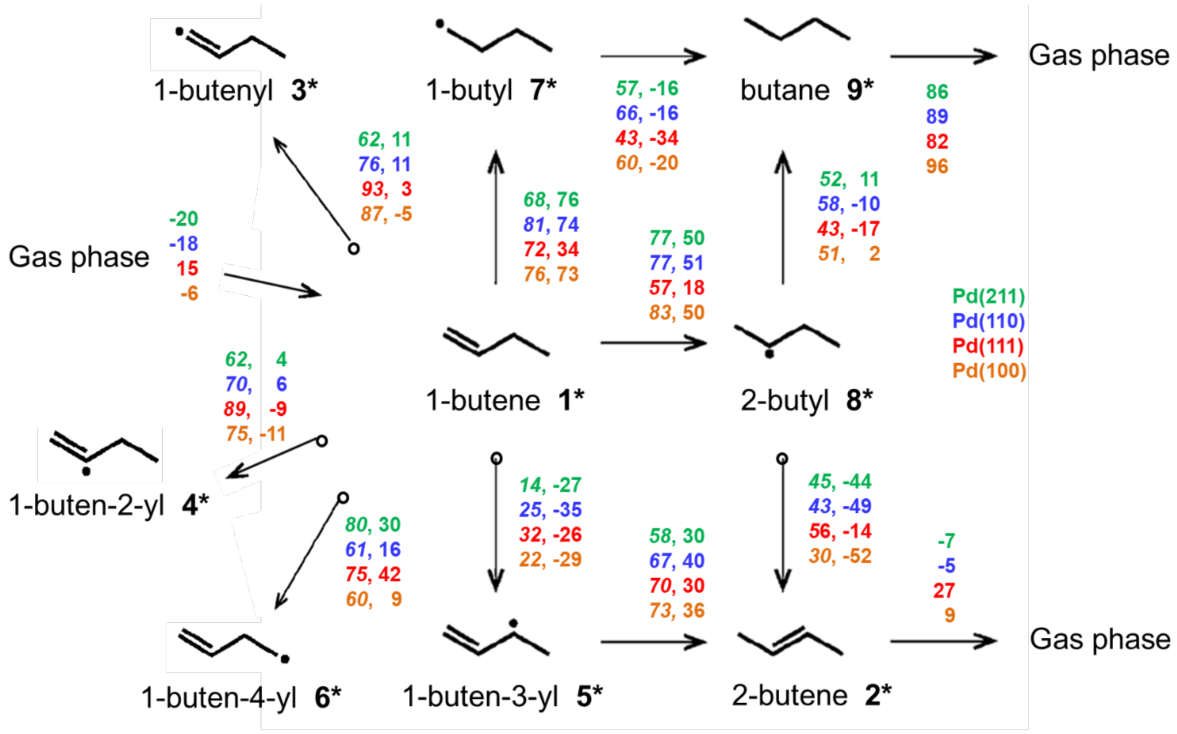

Supplementary Figure 2. Reaction network of the transformations of 1-butene on various Pd surfaces, at 0.5 ML H coverage. The labels of the reactions and various species are used throughout the main manuscript and the Supplementary Information. For each elementary step, activation free energy ΔG_a (first value, in italics), and the reaction free energy, ΔG_r (value in normal font). All energies in kJ mol^{-1}. Values calculated for the transformation on Pd(211) are given on top, in green, values for Pd(110) in blue, values for Pd(111) in red, and values for Pd(100) in orange.

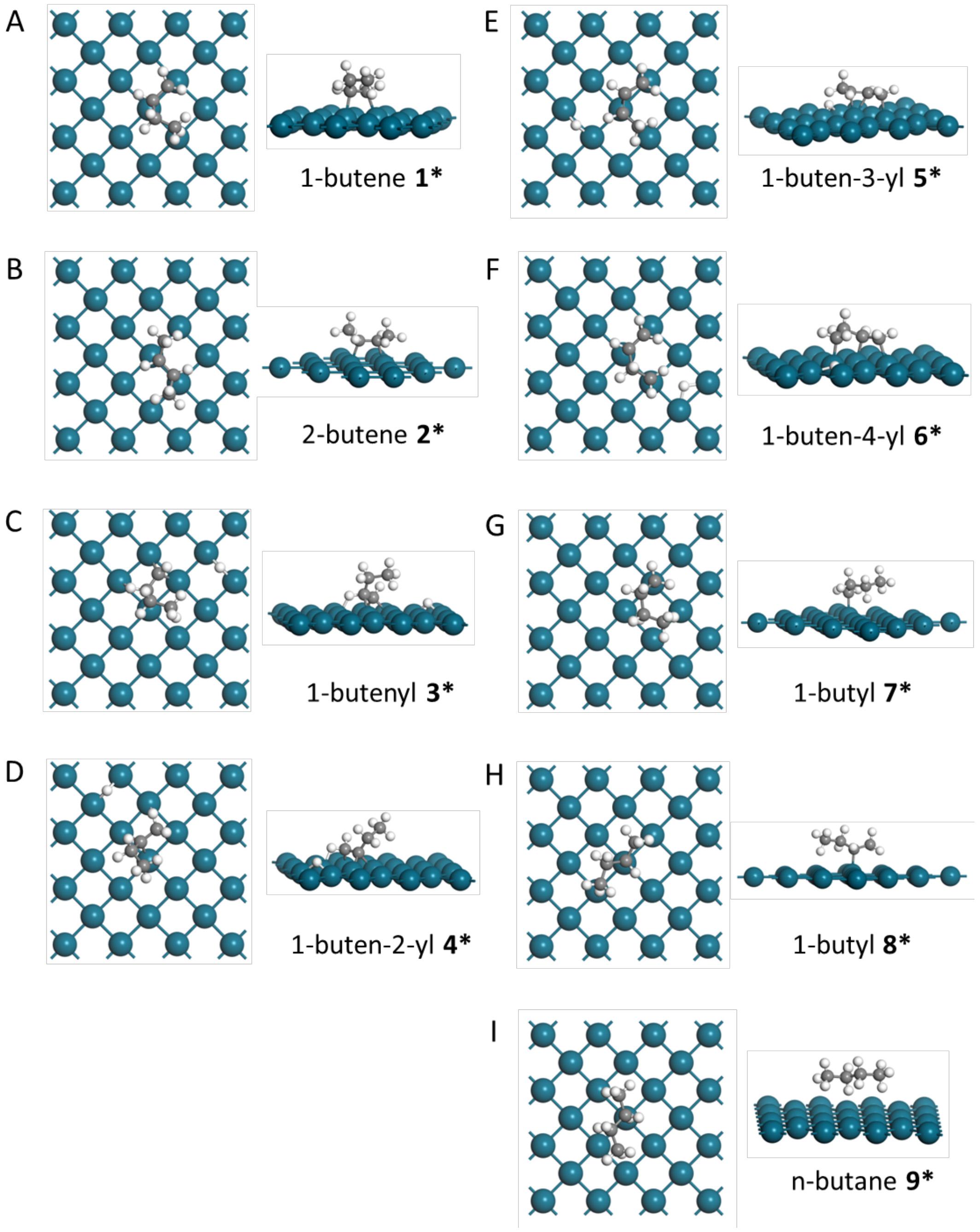

Supplementary Figure 3. Sketches of structures, top and side views, of various adsorption complexes on Pd(100). See the Supplementary Information of Ref. 12 for the analogous sketches regarding the surfaces Pd(111) and Pd(110).

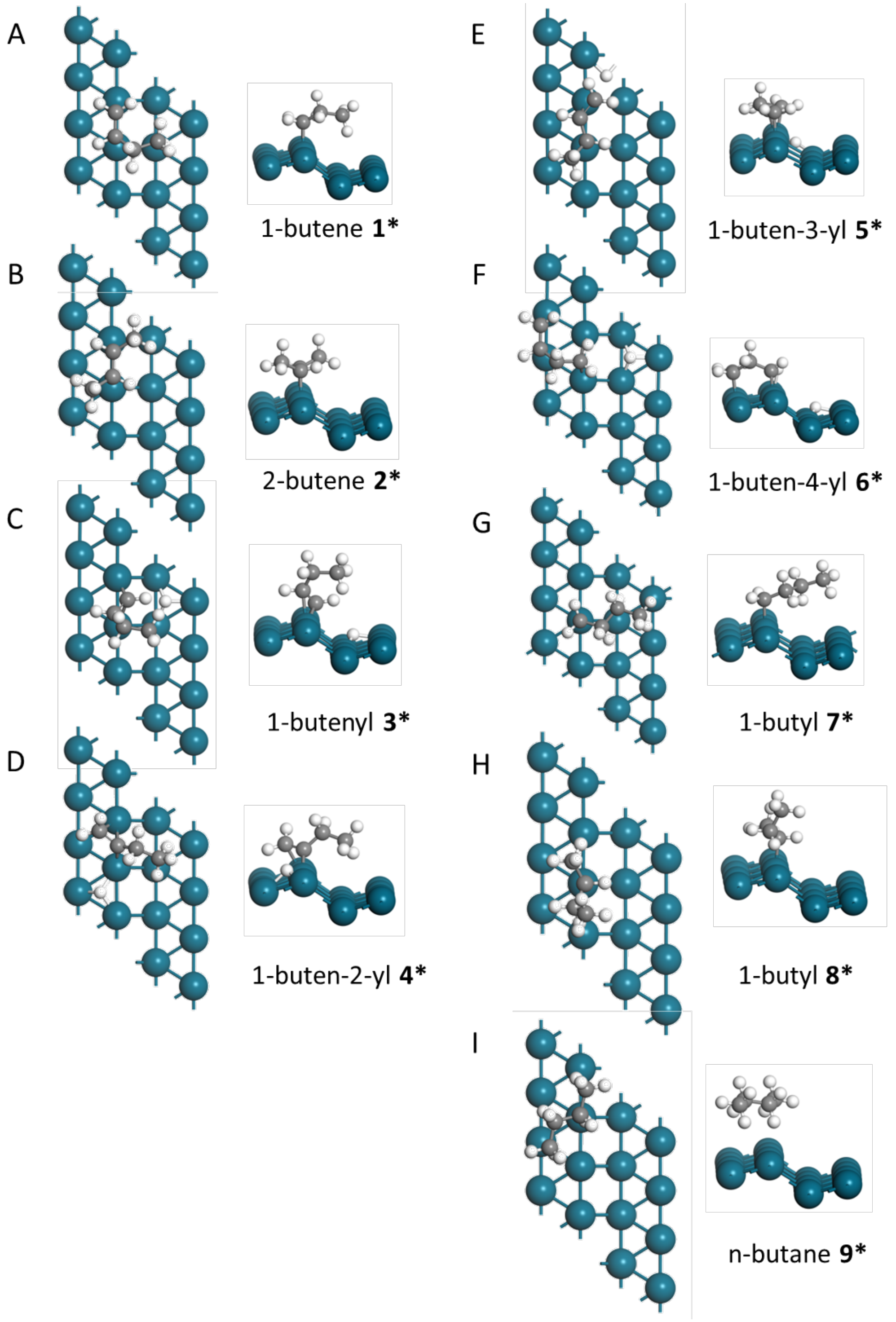

Supplementary Figure 4. Sketches of structures, top and side views, of various adsorption complexes on Pd(211). See the Supplementary Information of Ref. 12 for the analogous sketches regarding the surfaces Pd(111) and Pd(110).

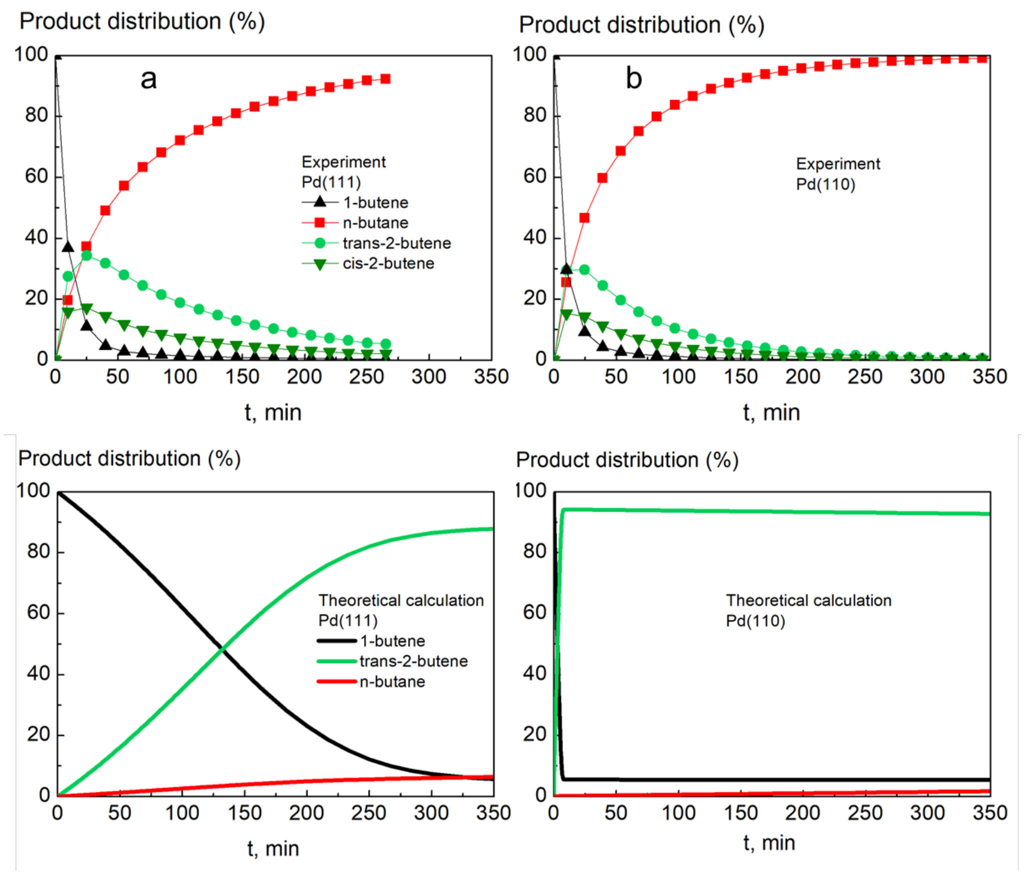

Supplementary Figure 5. Product distribution *vs*. reaction time for 1-butene hydrogenation at 373 K on various Pd surfaces. Left-hand column (a): Pd(111), right-hand column (b) Pd (110). Top row: experimental measurements. Initial reaction conditions: $P_{1\text{-butene}}$ – 5 mbar, P_{H2} – 10 mbar; Ar added to 1 bar at 373 K. Bottom row: microkinetic modeling with the initial conditions: $P_{1\text{-butene}}$ – 5 mbar, P_{H2} – 3.7 mbar. Note that 3.7 mbar of H_2 pressure were chosen based on previous work yielding a better agreement for single crystal surfaces ; see also Ref.12.

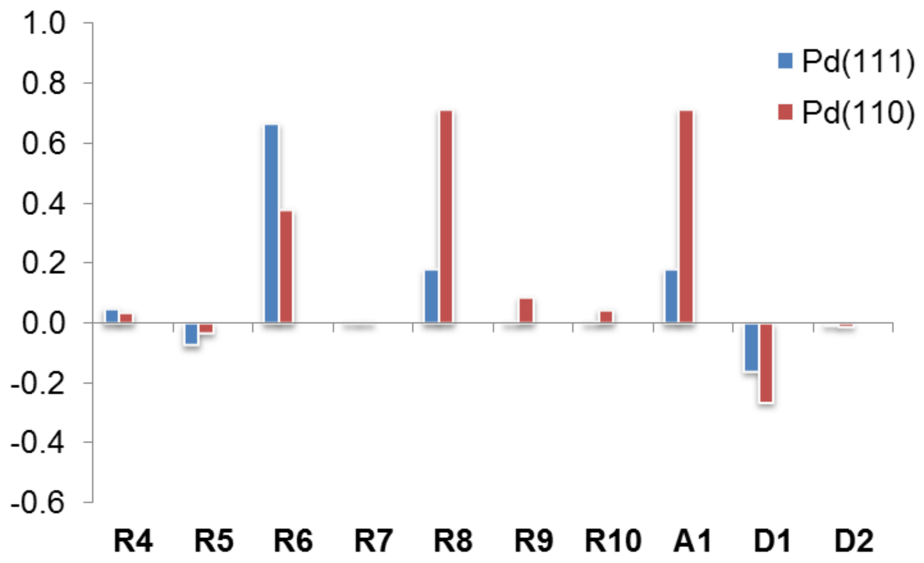

Supplementary Figure 6. Normalized sensitivity coefficients, NSC, for the formation of n-butane at 1% accumulation, determined for the surfaces Pd(111) and Pd(110) at T = 373 K, at a partial pressure of 1-butene at 5 mbar, and a partial pressure of H_2 at 3.7 mbar. For the labels, see Supplementary Figure 2 as well as Supplementary Tables 1 and 2.

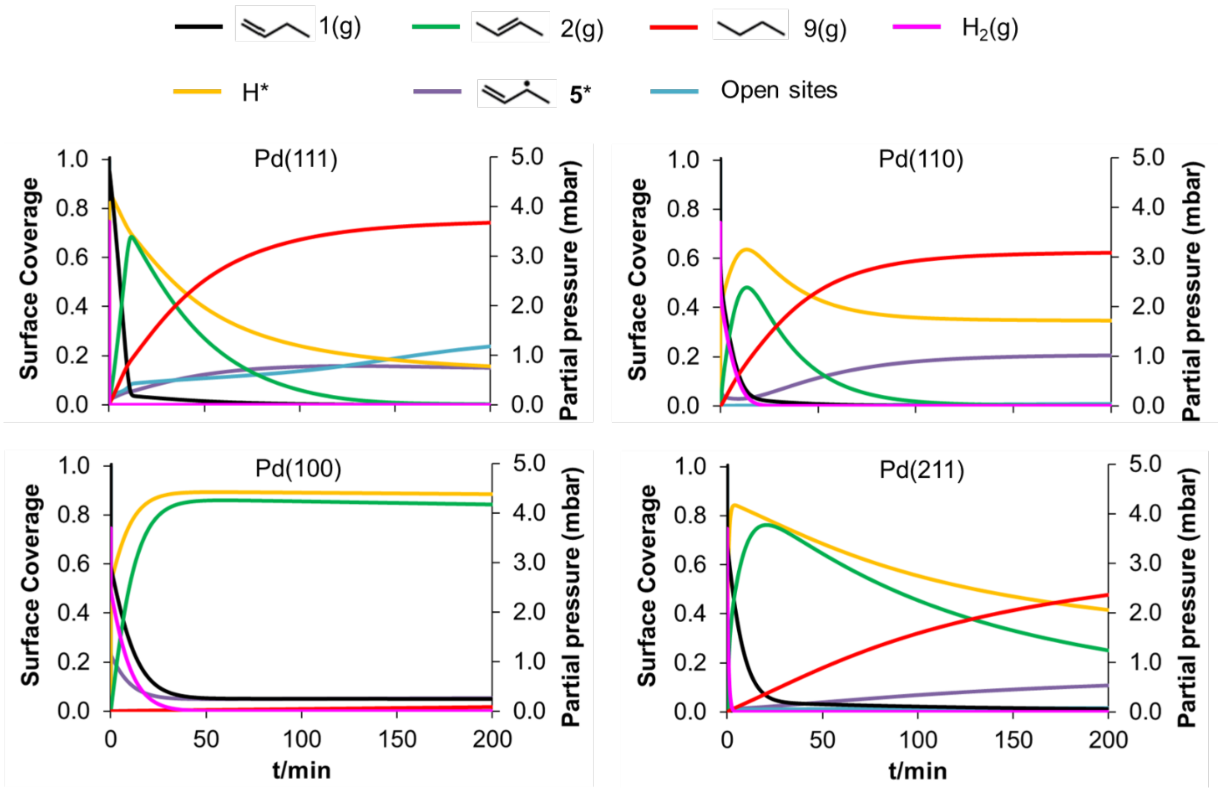

Supplementary Figure 7. Evolution in time of the coverage *θ* of adsorbed species, and of the partial pressure (in mbar) for gas phase species, (g). 3.7 mbar for initial H_2 pressure and 5 mbar for initial 1-butene pressure, at 373 K. Data obtained from micro-kinetic simulations with parameters determined for 0.5 ML H coverage.

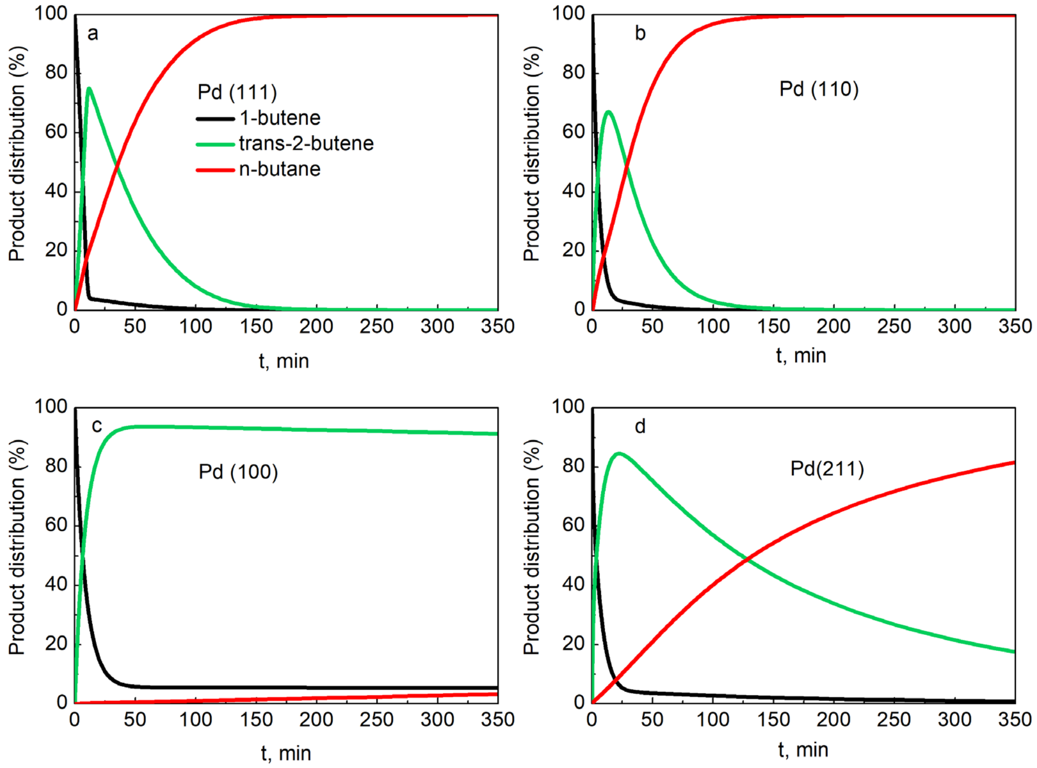

Supplementary Figure 8. Product distribution (in percent) of 1-butene transformation on the surfaces (a) Pd(111), (b) Pd(110), (c) Pd(100), and (d) Pd(211), from micro-kinetic simulations at 373 K with initial pressure 3.7 mbar for H_2 and 5 mbar for 1-butene. Color coding: black – 1-butene; green – trans-2-butene; red – n-butane.

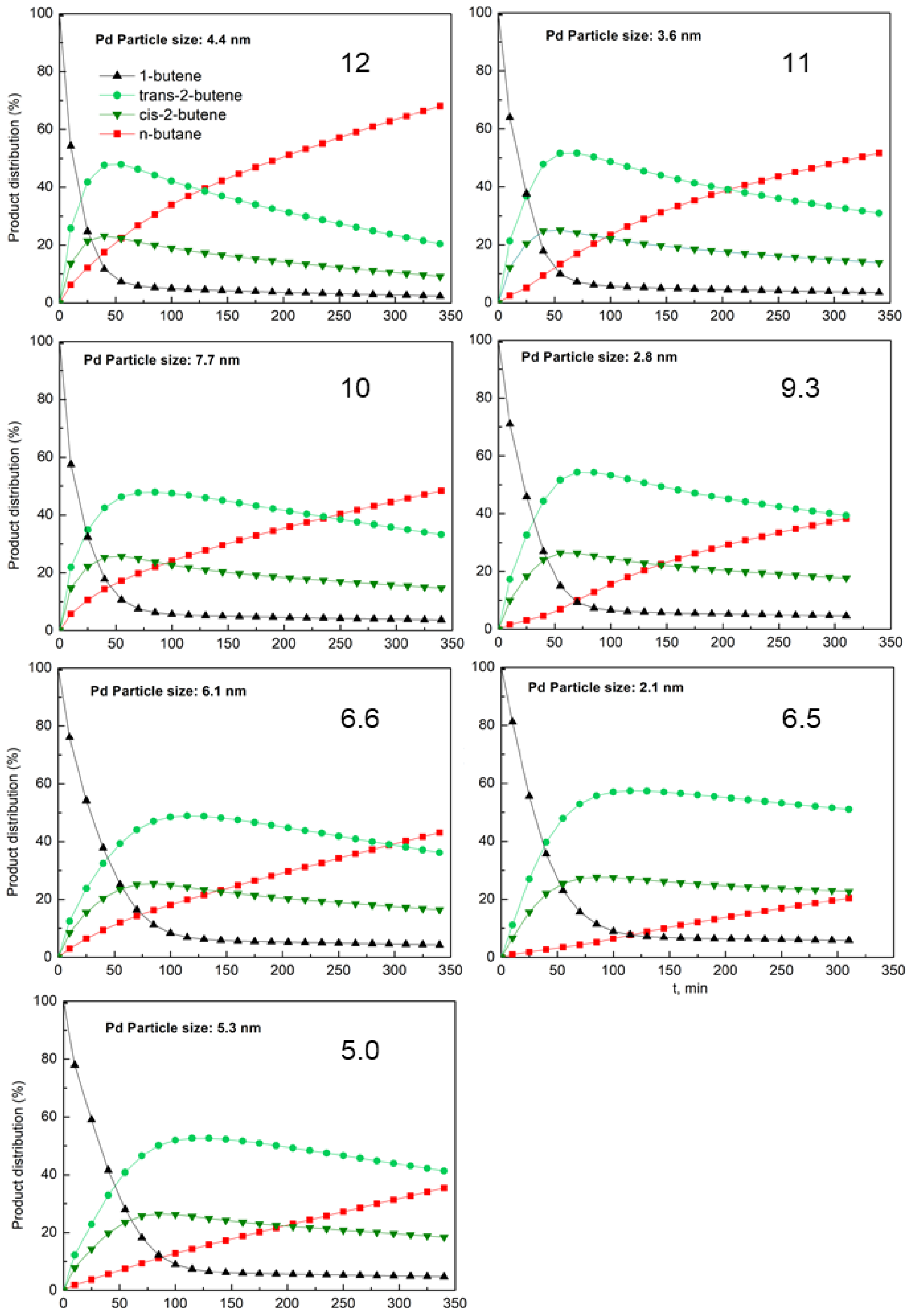

Supplementary Figure 9. Experimental product distribution vs. reaction time arranged by the total number of Pd surface atoms ($Pd_{s, tot}$, given in the upper right-hand corner of each panel) for 1-butene hydrogenation at 373 K on Pd nanocrystals supported by Al_2O_3/NiAl(110). The mean Pd particle size (from ~2 nm to ~8 nm). Reaction conditions: $P_{1\text{-butene}}$: 5 mbar; P_{H2}: 10 mbar; Ar added to 1 bar at 373 K. Values in large font indicate $Pd_{s, tot} \times 10^{14}$. Color coding: black – 1-butene; green – trans-2-butene; dark green – cis-2-butene; red – n-butane; cf. Supplementary Figure 10.

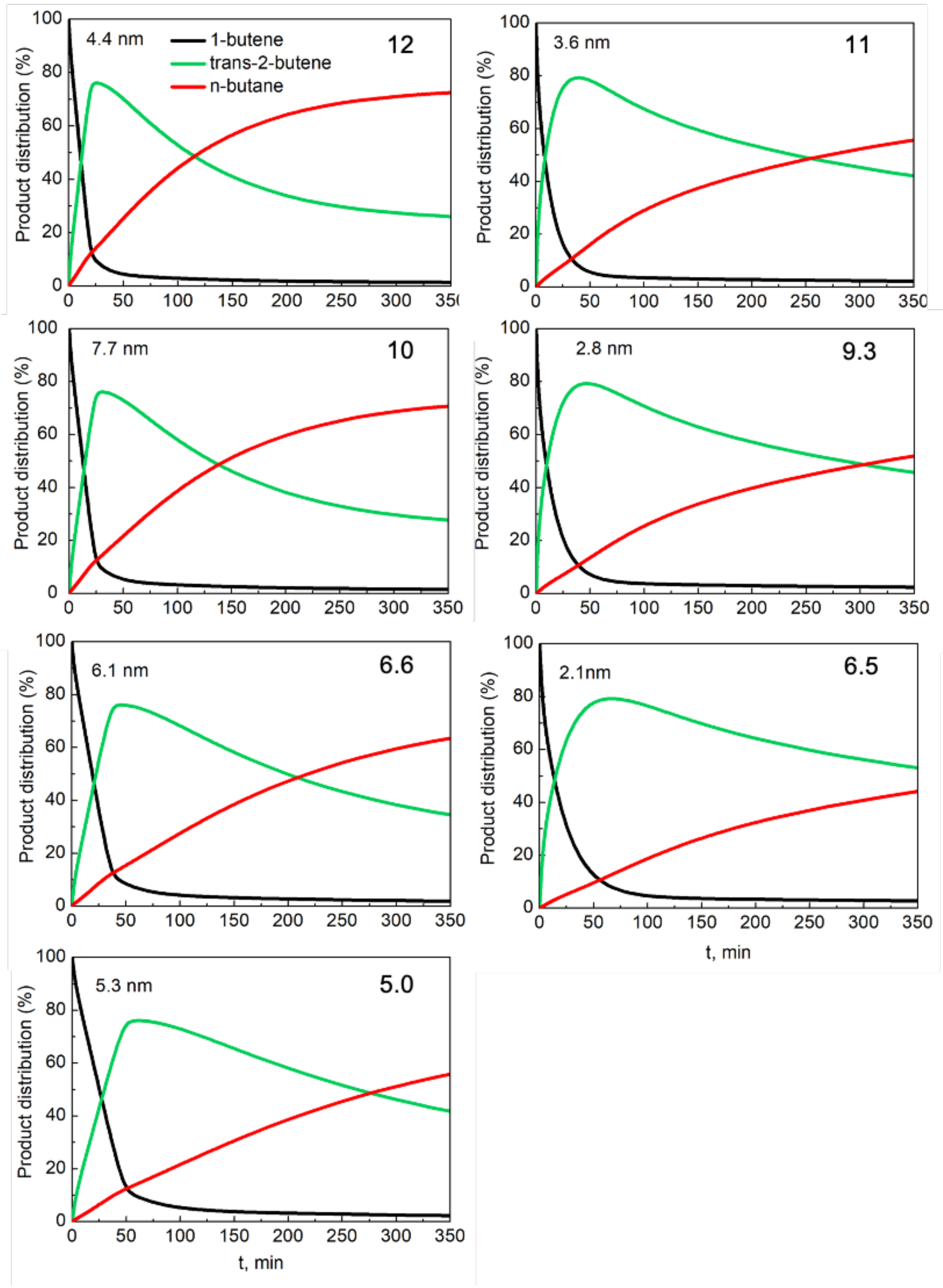

Supplementary Figure 10. Product distribution (in percent) arranged by the total number of Pd surface atoms ($Pd_{s,\ tot}$. given in the upper right-hand corner of each panel) for 1-butene transformation from microkinetic modeling on Pd nanoparticles of various sizes. The nanoparticles were modeled by mixing fixed ratios of surfaces as described in the main text. The micro-kinetic simulations were carried out at 373 K with initial pressure 3.7 mbar for H_2 and 5 mbar for 1-butene. Values in large font indicate $Pd_{s,\ tot}$, $\times 10^{14}$. Color coding as in Supplementary Figure 8.

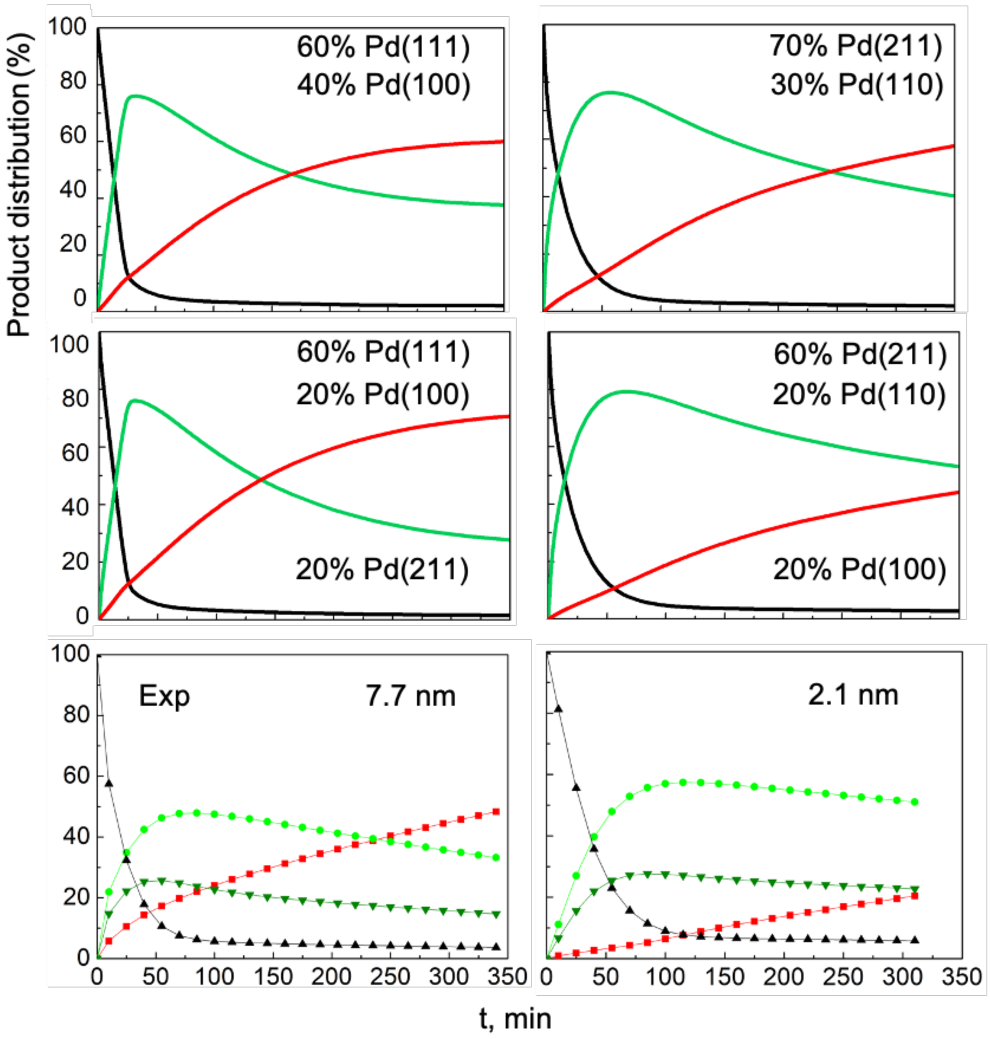

Supplementary Figure 11. Alternative mixed product distribution (in percent) vs time for 1-butene transformation from microkinetic modeling on Pd nanoparticles of 7.7 and 2.1 nm. The nanoparticles were modeled by mixing fixed ratios of surfaces as described in the main text. The micro-kinetic simulations were carried out at 373 K with initial pressure 3.7 mbar for H_2 and 5 mbar for 1-butene. Color coding as in Supplementary Figure 8.

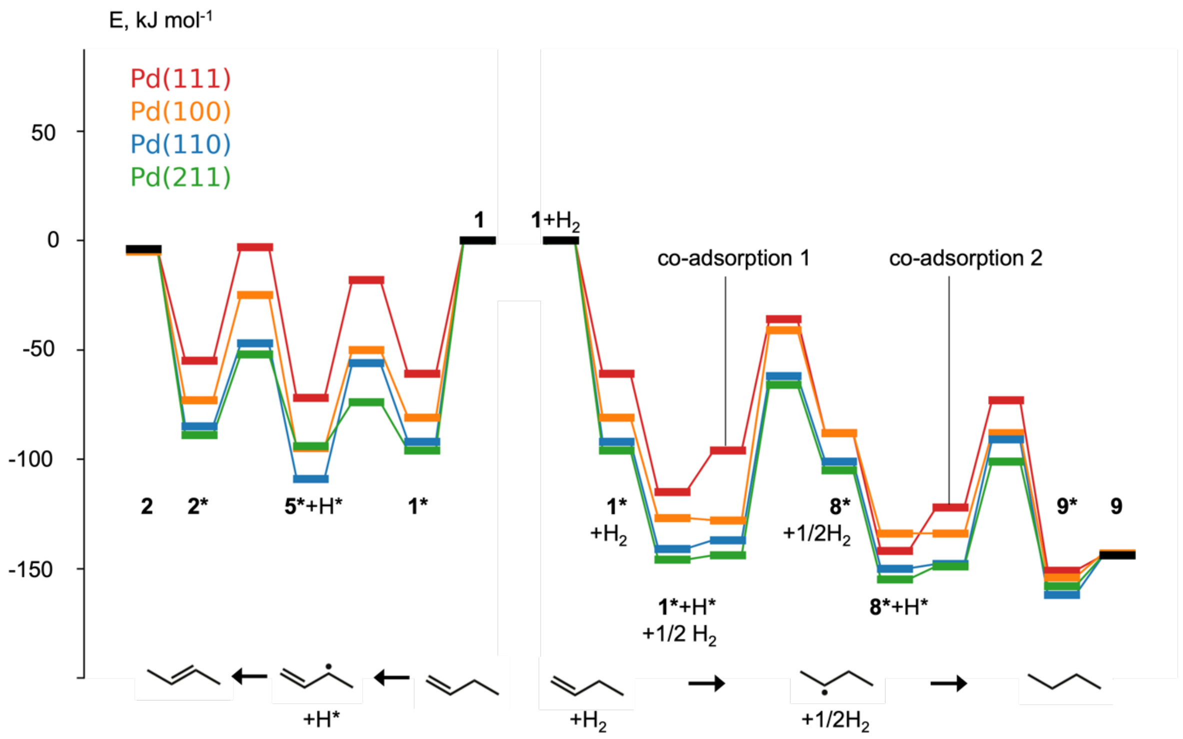

Supplementary Figure 12. Potential energy profile of isomerization vs. hydrogenation of 1-butene **1** at 0 K. In the isomerization process, going to the left from the central species **1**, we have a dehydrogenation step, leading to one H* co-adsorbed with **5***, which is consumed again when forming 2-butene **2***. In the hydrogenation process, going to the right, the energy reference is **1** + H_2. The adsorbate **1*** is formed, followed by forming ½ H_2* at formally infinite separation. Bringing both adsorbates together to an intermediate reaction pre-complex, labeled "co-adsorption 1", slightly raises the energy. Next follows the hydrogenation step **1*→8***, followed again by adding ½ H_2* at formally infinite distance, and forming a further reaction pre-complex, labeled "co-adsorption 2". In a second hydrogenation step on the right-hand side, adsorbed butane, **9***, is formed which desorbs easily.

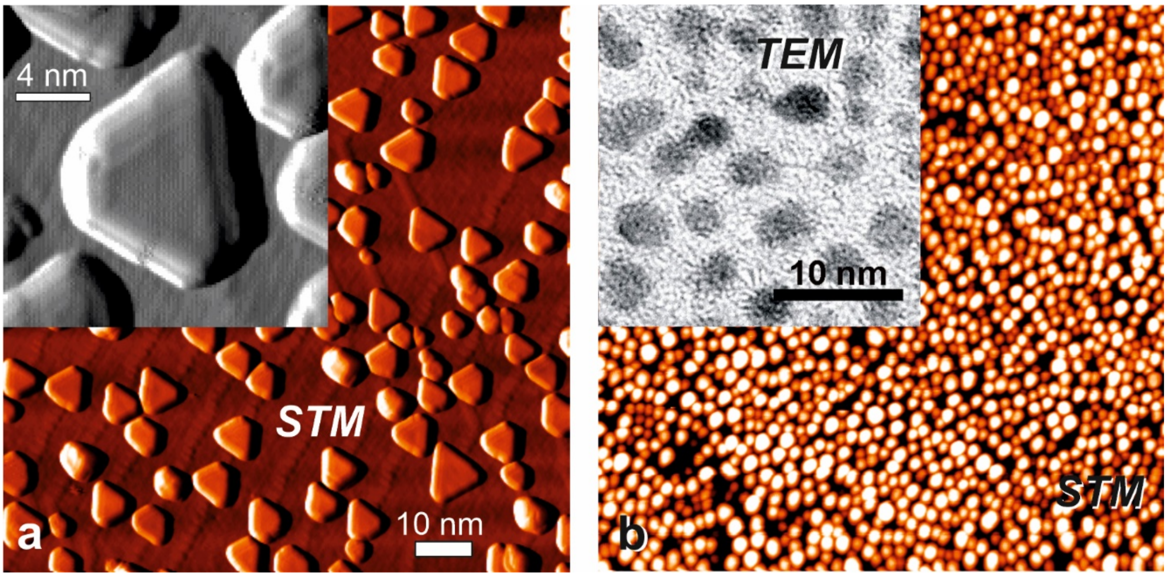

Supplementary Figure 13. STM images (100×100 nm^2) of Pd nanoparticles on Al_2O_3/NiAl(110), grown at 300 K (a) and at 90 K (b). The inset of the right-hand panel shows a transmission electron micrograph (TEM) at a higher magnification. Due to the "convolution" between the STM tip and the nanoparticle shape, the Pd particles appear larger and closer to each other (cf. STM and TEM in b). Accordingly, the mean particle size was calculated from the amount of evaporated Pd and the island density from STM.
STM images (a, b) adapted with permission from Ref. 32. Copyright (2003) Elsevier.
TEM image (inset in b) adapted with permission from Ref. 37. Copyright (2001) American Chemical Society.

Supplementary References

1 Kresse, G. & Hafner, J. Ab initio molecular-dynamics simulation of the liquid-metal–amorphous-semiconductor transition in germanium. *Phys. Rev. B* **49**, 14251-14269 (1994).

2 Kresse, G. & Furthmüller, J. Efficiency of ab-initio total energy calculations for metals and semiconductors using a plane-wave basis set. *Comput. Mater. Sci.* **6**, 15-50 (1996).

3 Perdew, J. P., Burke, K. & Ernzerhof, M. Generalized gradient approximation made simple. *Phys. Rev. Lett.* **77**, 3865-3868 (1996).

4 Perdew, J. P., Burke, K. & Ernzerhof, M. Comment on "generalized gradient approximation made simple"-reply. *Phys. Rev. Lett.* **80**, 891-891 (1998).

5 Wellendorff, J. *et al.* Density functionals for surface science: Exchange-correlation model development with Bayesian error estimation. *Phys. Rev. B* **85**, 235149-235172 (2012).

6 Blöchl, P. E. Projector augmented-wave method. *Phys. Rev. B* **50**, 17953-17979 (1994).

7 Kresse, G. & Joubert, D. From ultrasoft pseudopotentials to the projector augmented-wave method. *Phys. Rev. B* **59**, 1758-1775 (1999).

8 Methfessel, M. & Paxton, A. T. High-precision sampling for brillouin-zone integration in metals. *Phys. Rev. B* **40**, 3616-3621 (1989).

9 Mills, G., Jónsson, H. & Schenter, G. K. Reversible work transition state theory: Application to dissociative adsorption of hydrogen. *Surf. Sci.* **324**, 305-337 (1995).

10 Henkelman, G. & Jónsson, H. A dimer method for finding saddle points on high dimensional potential surfaces using only first derivatives. *J. Chem. Phys* **111**, 7010-7022 (1999).

11 Marchal, R., Genest, A., Krüger, S. & Rösch, N. Structure of Pd/Au alloy nanoparticles from a density functional theory-based embedded-atom potential. *J. Phys. Chem. C* **117**, 21810-21822 (2013).

12 Markova, V. K. *et al.* Catalytic transformations of 1-butene over palladium. A combined experimental and theoretical Study. *ACS Catal.* **8**, 5675-5685 (2018).

13 Horiuti, I. & Polanyi, M. Exchange reactions of hydrogen on metallic catalysts. *J. Chem. Soc. Faraday Trans* **30**, 1164-1172 (1934).

14 Campbell, C. T., Sprowl, L. H. & Árnadóttir, L. Equilibrium constants and rate constants for adsorbates: Two-dimensional (2D) ideal gas, 2D ideal lattice gas, and ideal hindered translator models. *J. Phys. Chem. C* **120**, 10283-10297 (2016).

15 Donaldson, D. J., Ammann, M., Bartels-Rausch, T. & Pöschl, U. Standard states and thermochemical kinetics in heterogeneous atmospheric chemistry. *J. Phys. Chem. A* **116**, 6312-6316 (2012).

16 Frisch, M. J. *et al. Gaussian 09, revision A.02*, Gaussian, Inc., Wallingford CT (2009).

17 Campbell, C. T. The degree of rate control: A powerful tool for catalysis research. *ACS Catal.* **7**, 2770-2779 (2017).

18 Buzzi-Ferraris, G. & Manenti, F. Library overview and recent advances in numerical methods. *Comput. Aided Chem. Eng.* **30**, 1312-1316 (2012).

19 Chorkendorff, I. & Niemantsverdriet, J. W. *Concepts of modern catalysis and kinetics Ch. 3.5.* (Wiley-VCH: Weinheim, Germany, 2005).

20 Resch, C., Berger, H. F., Rendulic, K. D. & Bertel, E. Adsorption dynamics for the system hydrogen/palladium and its relation to the surface electronic structure. *Surf. Sci.* **316**, L1105-L1109 (1994).

21 Conrad, H., Ertl, G. & Latta, E. E. Adsorption of hydrogen on palladium single crystal surfaces. *Surf. Sci.* **41**, 435-446 (1974).

22 Sainoo, Y. *et al.* Excitation of molecular vibrational modes with inelastic scanning tunneling microscopy processes: Examination through action spectra of cis-2-butene on Pd (110). *Phys. Rev. Lett.* **95**, 246102-246106 (2005).
23 Gautier, S., Steinmann, S. N., Michel, C., Fleurat-Lessard, P. & Sautet, P. Molecular adsorption at Pt(111). How accurate are DFT functionals? *Phys. Chem. Chem. Phys.* **17**, 28921-28930 (2015).
24 Campbell, C. T., Árnadóttir, L. & Sellers, J. R. Kinetic prefactors of reactions on solid surfaces. *Z. Phys. Chem.* **227**, 1435-1454. (2013).
25 Rupprechter, G., Dellwig, T., Unterhalt, H. & Freund, H. J. CO adsorption on Ni(100) and Pt(111) studied by infrared–visible sum frequency generation spectroscopy: Design and application of an SFG-compatible UHV–high-pressure reaction cell. *Top. Catal.* **15**, 19-26 (2001).
26 Rupprechter, G. Surface vibrational spectroscopy from ultrahigh vacuum to atmospheric pressure: adsorption and reactions on single crystals and nanoparticle model catalysts monitored by sum frequency generation spectroscopy. *Phys. Chem. Chem. Phys.* **3**, 4621-4632 (2001).
27 Bäumer, M. & Freund, H.-J. Metal deposits on well-ordered oxide films. *Prog. Surf. Sci.* **61**, 127-198 (1999).
28 Freund, H.-J., Bäumer, M. & Kuhlenbeck, H. Catalysis and surface science: What do we learn from studies of oxide-supported cluster model systems? *Adv. Catal.* **45**, 333-384 (2000).
29 Rupprechter, G. Sum frequency generation and polarization–modulation infrared reflection absorption spectroscopy of functioning model catalysts from ultrahigh vacuum to ambient pressure. *Adv. Catal.* **51**, 133-263 (2007).
30 Silvestre-Albero, J., Rupprechter, G. & Freund, H.-J. Atmospheric pressure studies of selective 1,3-butadiene hydrogenation on well-defined Pd/Al_2O_3/NiAl(110) model catalysts: Effect of Pd particle size. *J. Catal.* **240**, 58-65 (2006).
31 Freund, H. J. Adsorption of gases on complex solid surfaces. *Angew. Chem. Int. Ed.* **36**, 452-475 (1997).
32 Freund, H.-J. *et al.* Preparation and characterization of model catalysts: From ultrahigh vacuum to in situ conditions at the atomic dimension. *J. Catal.* **216**, 223-235 (2003).
33 Hansen, K. H. *et al.* Palladium nanocrystals on Al2O3: structure and adhesion energy. *Phys. Rev. Lett.* **83**, 4120-4123 (1999).
34 Shaikhutdinov, S. *et al.* Interaction of oxygen with palladium deposited on a thin alumina film. *Surf. Sci.* **501**, 270-281 (2002).
35 Wolter, K., Seiferth, O., Kuhlenbeck, H., Bäumer, M. & Freund, H. J. Infrared spectroscopic investigation of CO adsorbed on Pd aggregates deposited on an alumina model support. *Surf. Sci.* **399**, 190-198 (1998).
36 Dellwig, T., Rupprechter, G., Unterhalt, H. & Freund, H. J. Bridging the pressure and materials gaps: High pressure sum frequency generation study on supported Pd nanoparticles. *Phys. Rev. Lett.* **85**, 776-779 (2000).
37 Unterhalt, H., Rupprechter, G. & Freund, H.-J. Vibrational sum frequency spectroscopy on Pd(111) and supported Pd nanoparticles: CO adsorption from ultrahigh vacuum to atmospheric pressure. *J. Phys. Chem. B* **106**, 356-367 (2002).
38 Yudanov, I. V. *et al.* CO adsorption on Pd nanoparticles: Density functional and vibrational spectroscopy studies. *J. Phys. Chem. B* **107**, 255-264 (2003).
39 Heemeier, M. *et al.* On the thermal stability of metal particles supported on a thin alumina film. *Surf. Sci.* **523**, 103-110 (2003).
40 Dobrezberger, K. *et al.* Hydrogenation on palladium nanoparticles supported by graphene nanoplatelets. *J. Phys. Chem. C* **124**, 23674-23682 (2020).

41 Frank, M. & Bäumer, M. From atoms to crystallites: Adsorption on oxide-supported metal particles. *Phys. Chem. Chem. Phys.* **2**, 3723-3737 (2000).
