# Supplementary material for: The origin of the particle-size-dependent selectivity in 1-butene isomerization and hydrogenation on Pd/Al2O3 catalysts
Source: Nat Commun. 2021 Oct 20;12:6098. doi: 10.1038/s41467-021-26411-8 (PMC8528898; doi:10.1038/s41467-021-26411-8)
Supplement: Supplementary file 2 — Reporting Summary [file 41467_2021_26411_MOESM2_ESM.pdf]

nature research

Corresponding author(s): NCOMMS-21-11808-D

Last updated by author(s): Sep 22, 2021

Reporting Summary

Nature Research wishes to improve the reproducibility of the work that we publish. This form provides structure for consistency and transparency in reporting. For further information on Nature Research policies, see our Editorial Policies and the Editorial Policy Checklist.

Statistics

For all statistical analyses, confirm that the following items are present in the figure legend, table legend, main text, or Methods section.

n/a	Confirmed	
☐	☒	The exact sample size (n) for each experimental group/condition, given as a discrete number and unit of measurement
☐	☒	A statement on whether measurements were taken from distinct samples or whether the same sample was measured repeatedly
☒	☐	The statistical test(s) used AND whether they are one- or two-sided<br>*Only common tests should be described solely by name; describe more complex techniques in the Methods section.*
☐	☒	A description of all covariates tested
☒	☐	A description of any assumptions or corrections, such as tests of normality and adjustment for multiple comparisons
☒	☐	A full description of the statistical parameters including central tendency (e.g. means) or other basic estimates (e.g. regression coefficient) AND variation (e.g. standard deviation) or associated estimates of uncertainty (e.g. confidence intervals)
☒	☐	For null hypothesis testing, the test statistic (e.g. F, t, r) with confidence intervals, effect sizes, degrees of freedom and P value noted<br>*Give P values as exact values whenever suitable.*
☒	☐	For Bayesian analysis, information on the choice of priors and Markov chain Monte Carlo settings
☒	☐	For hierarchical and complex designs, identification of the appropriate level for tests and full reporting of outcomes
☒	☐	Estimates of effect sizes (e.g. Cohen's d, Pearson's r), indicating how they were calculated

Our web collection on statistics for biologists contains articles on many of the points above.

Software and code

Policy information about availability of computer code

Data collection	Experimental data acquired using a home-built catalytic reactor and gas chromatographic analysis (HP with flame ionization detector)
Data analysis	MS Office Professional Plus 2019 (Excel), OriginPro 2020 V9.7.0.185

For manuscripts utilizing custom algorithms or software that are central to the research but not yet described in published literature, software must be made available to editors and reviewers. We strongly encourage code deposition in a community repository (e.g. GitHub). See the Nature Research guidelines for submitting code & software for further information.

Data

Policy information about availability of data

All manuscripts must include a data availability statement. This statement should provide the following information, where applicable:
- Accession codes, unique identifiers, or web links for publicly available datasets
- A list of figures that have associated raw data
- A description of any restrictions on data availability

The data that support the findings of this study are available from the corresponding author upon reasonable request.

Field-specific reporting

Please select the one below that is the best fit for your research. If you are not sure, read the appropriate sections before making your selection.

☐ Life sciences ☐ Behavioural & social sciences ☒ Ecological, evolutionary & environmental sciences

For a reference copy of the document with all sections, see nature.com/documents/nr-reporting-summary-flat.pdf

Ecological, evolutionary & environmental sciences study design

All studies must disclose on these points even when the disclosure is negative.

Study description	The selectivity of 1-butene hydrogenation/isomerization on Pd catalysts was examined by combining well-defined model catalysts, atmospheric pressure reactor kinetics, DFT calculations and microkinetic modeling. This enabled to rationalize the well-known particle size dependence based on the abundance and the specific properties of the contributing surface facets.
Research sample	Pd nanoparticles supported on ultrathin Al2O3 film grown on NiAl(110)
Sampling strategy	One sample for each particle size. 7 different particle sizes. Substrate size of NiAl (0.78 cm^2).
Data collection	A.G. contributed to the interpretation and supervised the microkinetic modeling. J.S.-A. performed the experiments. W.Q.L. carried out the microkinetic modeling. A.G. and N.R. wrote the modelling part of the manuscript, J.S.-A. and G.R. wrote the experimental draft of the manuscript, which was finally revised by all authors. N.R. and G.R. supervised the research and acquired the funding.
Timing and spatial scale	Experimental data and computational modelling carried out 2007-2020, based on several research visits and project meetings.
Data exclusions	none
Reproducibility	Confirmed by repeated measurements and computations.
Randomization	*Describe how samples/organisms/participants were allocated into groups. If allocation was not random, describe how covariates were controlled. If this is not relevant to your study, explain why.*
Blinding	n.a.

Did the study involve field work? ☐ Yes ☒ No

Reporting for specific materials, systems and methods

We require information from authors about some types of materials, experimental systems and methods used in many studies. Here, indicate whether each material, system or method listed is relevant to your study. If you are not sure if a list item applies to your research, read the appropriate section before selecting a response.

Materials & experimental systems

n/a	Involved in the study	
☒	☐	Antibodies
☒	☐	Eukaryotic cell lines
☒	☐	Palaeontology and archaeology
☒	☐	Animals and other organisms
☒	☐	Human research participants
☒	☐	Clinical data
☒	☐	Dual use research of concern

Methods

n/a	Involved in the study	
☒	☐	ChIP-seq
☒	☐	Flow cytometry
☒	☐	MRI-based neuroimaging
